# Thalamus and consciousness: a systematic review on thalamic nuclei associated with consciousness

**DOI:** 10.3389/fneur.2025.1509668

**Published:** 2025-06-18

**Authors:** Martina Cacciatore, Francesca Giulia Magnani, Filippo Barbadoro, Camilla Ippoliti, Mario Stanziano, Letizia Clementi, Anna Nigri, Lorenzo Nanetti, Silvia Marino, Fabio La Porta, Lucia Francesca Lucca, Francesco Prada, Matilde Leonardi

**Affiliations:** ^1^SC Neurologia, Salute Pubblica, Disabilità, Fondazione IRCCS Istituto Neurologico Carlo Besta, Milan, Italy; ^2^Neuroradiology Unit, Fondazione IRCCS Istituto Neurologico "Carlo Besta", Milan, Italy; ^3^Department of Neurosciences “Rita Levi Montalcini”, University of Turin, Turin, Italy; ^4^Department of Neuroradiology, Fondazione IRCCS Istituto Neurologico Carlo Besta, Milan, Italy; ^5^Medical Genetics and Neurogenetics Unit, Fondazione IRCCS Istituto Neurologico Carlo Besta Milan, Milan, Italy; ^6^IRCCS Centro Neurolesi "Bonino Pulejo", Messina, Italy; ^7^IRCCS Istituto delle Scienze Neurologiche di Bologna, Bologna, Italy; ^8^Intensive Rehabilitation Unit, S. Anna Institute, Crotone, Italy; ^9^Department of Neurological Surgery, Fondazione IRCCS Istituto Neurologico "C. Besta", Milan, Italy; ^10^Acoustic Neuroimaging and Therapy Laboratory (ANTY-Lab), Fondazione IRCCS Istituto Neurologico Carlo Besta, Milan, Italy

**Keywords:** thalamus, consciousness, centromedian-parafascicular, neuromodulation, arousal, wakefulness, disorders of consciousness

## Abstract

**Introduction:**

Consciousness relies on both cortical and subcortical structures and their feedforward and feedback pathways. Within this framework, the thalamus represents a relay station enabling the transmission, reception, and integration of information. However, it is divided into several nuclear groups each composed of functionally heterogeneous nuclei, and, to date, an agreement on which nuclei are most involved in the generation, maintenance, and modulation of consciousness is still lacking.

**Methods:**

To fill this gap, we performed a systematic review by querying 5 electronic databases (MEDLINE/Pubmed, Scopus, EMBASE, Web of Science, and Cinahl Complete) about studies published in the last 20 years exploring the relationship between specific thalamic nuclei/nuclear groups and consciousness. For each included study, we extracted data supporting (i.e., positive evidence) or not (i.e., negative evidence) the relationship between a specific nucleus/nuclear group and the consciousness.

**Results:**

167 articles were included leading to 346 pieces of evidence of which 284 were positive. Most of the retrieved positive evidence pertained to the intralaminar nuclear group, followed by the mediodorsal and ventral nuclear groups. Furthermore, when considering the specific nuclei within the intralaminar nuclear group, results highlighted the centromedian-parafascicular complex (CM-Pf) as the nucleus most related to consciousness. Despite the high heterogeneity characterizing the adopted methodologies (e.g., brain stimulation, anesthesia, brain damage), as well as the study population (e.g., either healthy and pathological humans or animals) across studies, the greatest amount of evidence supported a key role of CM-Pf for the generation, modulation, and maintenance of the level of consciousness.

**Discussion:**

Though there is more research on the role of intralaminar nuclei, there is proportionally more positive evidence supporting these nuclei (particularly the CM-Pf) as key nodes in the network underlying consciousness compared to other thalamic nuclei. These results support ongoing therapeutic approaches to disorders of consciousness by reinforcing the rationale behind brain stimulation targeting CM-Pf and paving the way for other potential candidates for targeted interventions.

## 1 Introduction

Studying consciousness is a major challenge in neuroscience, and a shared definition is still lacking (1, 2). From a classical neurological perspective, consciousness encompasses two main components: wakefulness (i.e., the level of consciousness) and awareness (i.e., the content of consciousness) (1, 3). Whilst wakefulness refers to being awake, awareness is characterized by conscious access to a specific piece of information, thus representing the subjective experience (3). Moreover, several authors (4) highlighted the role of arousal as a key component in the generation of consciousness. Specifically, arousal reflects the overall state of alertness, and it may be considered as the background condition that enables consciousness by ensuring adequate excitability of the neuronal substrate of consciousness, without being directly involved in specifying conscious contents (5).

Similarly, there is still much debate on identifying the neuronal correlates of consciousness [NCCs; Koch et al. (6)] defined as the minimum set of neuronal mechanisms sufficient to be conscious. Although we are far from a univocal identification of the NCC, consciousness relies on both cortical and subcortical structures with their feedforward and feedback pathways. Specifically, consciousness is thought to be supported by “reentrant” activity with continuous interactions from deep layers of subcortical areas to superficial and middle layers of high-order cortical areas (i.e., feedforward pathways) and vice versa (i.e., feedback pathways) (7–9). Within this framework, the thalamus plays a key role as a relay station allowing the transmission, reception, and integration of a large variety of cortical and subcortical information.

The thalamus has been defined as a central “miniature-map” of the brain, where each cortical area is represented in specific thalamic nuclei (10). The identification of these nuclei depends on the thalamic parcellation adopted as reference (e.g., topographical, cytoarchitectural, and functional) (10–13). Based on the topographical features and the excitatory and inhibitory nature of the projections, the thalamus has been traditionally divided into dorsal and ventral parts, respectively (10, 14). The dorsal thalamus encompasses several nuclei gathered in nine nuclear groups (i.e., the anterior, mediodorsal; MD, lateral, ventral, intralaminar, midline, posterior, and medial and lateral geniculate bodies). Instead, the ventral thalamus encompasses the reticular nucleus (TRN) only (15), that comprises GABAergic neurons reciprocally connecting cortical and subcortical structures and representing the inhibitory control over other thalamic nuclei and thalamo-cortical connections (16, 17). Several studies demonstrated that the TRN participates in regulating sleep/wake cycle (18–20), absence seizures (21), and it is a target of anesthetic drugs (22), thus suggesting its crucial role in the mechanisms underlying wakefulness, arousal, and consciousness. Despite the well-recognized role of TRN in maintaining arousal and wakefulness by representing the major component modulating the synchronization of thalamo-cortical networks (23), the dorsal thalamic nuclei deserve attention as well. Indeed, by considering the projection to the higher-order cortical areas, dorsal thalamic nuclei may be functionally classified into (i) relay nuclei, also known as “specific nuclei” due to their projections to the primary motor and sensory cortices; (ii) association nuclei, receiving inputs from the sensorimotor cortex and projecting to both association and limbic cortical areas, and (iii) intralaminar and midline nuclei, also known as “non-specific nuclei” due to their widespread projections to the cerebral cortex, striatum, and basal ganglia (10, 13, 14).

The non-specific thalamic nuclei representing the central parts of the forebrain arousal system are responsible for the overall level of cortical excitability and, therefore, have been linked to the level of consciousness (24); moreover, they have been further divided depending on the functions they contributed to. Specifically, the midline nuclear group is conventionally divided into a ventral part involving the nucleus reuniens (Re) and linked to several cognitive functions [e.g., working memory and executive functions; Vertes et al. (14)] and a dorsal part encompassing paraventricular (Pv) and paratenial (Pt) nuclei which are related to behaviors requiring elevated wakefulness [e.g., feeding or fear; Ren et al. (25)]. On the other hand, the intralaminar nuclei are subdivided into rostral (i.e., paracentral; Pc, central lateral; CL, and central medial; CeM) and caudal (i.e., centromedian-parafascicular complex; CM-Pf) nuclei. It is well known that being the intralaminar nuclei a termination site of the Ascending Reticular Activating System (ARAS) (26), they have a pivotal role in regulating the cortical arousal state and, over the years, a growing number of studies investigated their functioning focusing primarily on the CL and CM-Pf nuclei due to their role in promoting cortical excitation and influencing feedforward and feedback pathways (27–29). The role of non-specific thalamic nuclei for consciousness was also supported by the studies on severely brain-injured patients with Disorders of Consciousness (DOC) (30, 31), and current research provided remarkable findings about the use of intralaminar Deep Brain Stimulation (DBS) targeting the CL and CM-Pf as a potential neuromodulatory intervention to boost the level of consciousness of these patients (32).

However, it is worth noting that, although the non-specific nuclei have been historically identified as pivotal structures for consciousness, available evidence reported the implication of relay/specific and association thalamic nuclei as well. Accordingly, Schiff et al. (31) pointed out that along with the intralaminar nuclei, the MD, ventral anterior (VA), ventral lateral (VL), and inferior pulvinar (PULi) nuclei (together defined as central thalamus) also possess anatomical and functional features suitable to support consciousness. Indeed, they are interconnected with the basal forebrain systems controlling cortical activity, thus, they may be part of the large-scale cerebral dynamics underlying consciousness (31). Their role in supporting consciousness is highlighted by several studies investigating their activity during anesthesia (33) and in pathological populations manifesting DOC (34, 35) or absence seizures (36).

Taken together, the above-reported evidence highlighted the thalamus as a key structure for the regulation of the overall state of arousal, wakefulness, and consciousness. Although the role of the different nuclei has been proved by adopting different methodologies on different populations, an agreement on which nucleus (if any) most contributed to consciousness is still lacking. To fill this gap, we performed a systematic review of the studies published in the last 20 years exploring the relationship between specific thalamic nuclei and consciousness, arousal, and wakefulness (as these two last components contribute to consciousness). In other words, the present systematic review aimed to pool together the heterogeneous existing evidence concerning the relation between specific thalamic nuclei and consciousness to determine which thalamic nuclei are most related to it. Whether it would be possible identifying the thalamic nucleus playing a pivotal role in generating, modulating, and maintaining consciousness, it would have important clinical consequences, especially for targeting the most appropriate thalamic region to boost the level of consciousness through neuromodulation in clinical population suffering from DOC.

## 2 Methods

The present systematic review followed the Preferred Reporting Items for Systematic Reviews and Meta- Analyses guidelines [PRISMA; Moher et al. (37)] to search and extract eligible studies.

### 2.1 Search strategy

Relevant studies were identified by searching the following electronic databases: MEDLINE/Pubmed, Scopus, EMBASE, Web of Science, and Cinahl Complete. The search was narrowed to the title, abstract, or keywords of original published studies, and a tailored search strategy was developed for each database according to their thesaurus (see Supplementary material for terms combinations). The publication dates were initially set from the beginning of 2003 until March 2023 and then updated until the 15th of July 2024. All the searches were limited to articles published in English. Both animal and human studies have been considered. Moreover, the reference list of the topic-relevant reviews was assessed to identify further eligible studies to be added.

The duplicate deletion was performed by a bibliographic management software (Mendeley; https://www.mendeley.com, accessed on 15th of July 2024), and the records were imported in a customized Excel spreadsheet including the title, abstract, and record information for each article.

### 2.2 Selection criteria

Studies were eligible if they met all the following criteria: (i) to be a research article (i.e., excluding reviews, book chapters, and theoretical studies not reporting experimental data), (ii) either measuring or manipulating consciousness, arousal, or wakefulness, and (iii) presenting evidence in favor or against a relationship between one of the above-mentioned functions and a specific thalamic nucleus or nuclear group. We did not apply any restrictions on the techniques adopted to manipulate and measure the function of interest. Consequently, we included studies manipulating consciousness, arousal, and wakefulness through either brain-modulatory techniques (e.g., electrical, chemogenetic, and optogenetic stimulation), or anesthesia protocols, as well as studies measuring the function of interest in pathological populations. No restrictions on the study population were adopted, including both studies on animals and humans, being either healthy or pathological (e.g., epilepsy, severe acquired brain injuries leading to DOC).

### 2.3 Screening and data extraction

Eligibility assessment of search results was performed independently by three raters using a three-step procedure.

In the first step, articles were screened by title, abstract, and keywords by adopting the following assessment scale: 0 = excluded (i.e., the article did not meet the inclusion criteria); 1 = included (i.e., the article matched the inclusion criteria); 2 = doubt (i.e., the article required the intervention of a third rater). The agreement between the raters was computed [Cohen K (38)]. In the second step, the full text of the included articles was analyzed using a similar rating (i.e., 0 = excluded, 1 = included). In both steps, a third rater with senior experience in the topic reviewed the articles to reach a consensus in cases of discrepancy between the raters.

In the final step, raters independently extracted data from the included studies using a custom-built Excel data extraction sheet, pilot-tested on fifteen randomly selected included studies.

**Figure 1:**
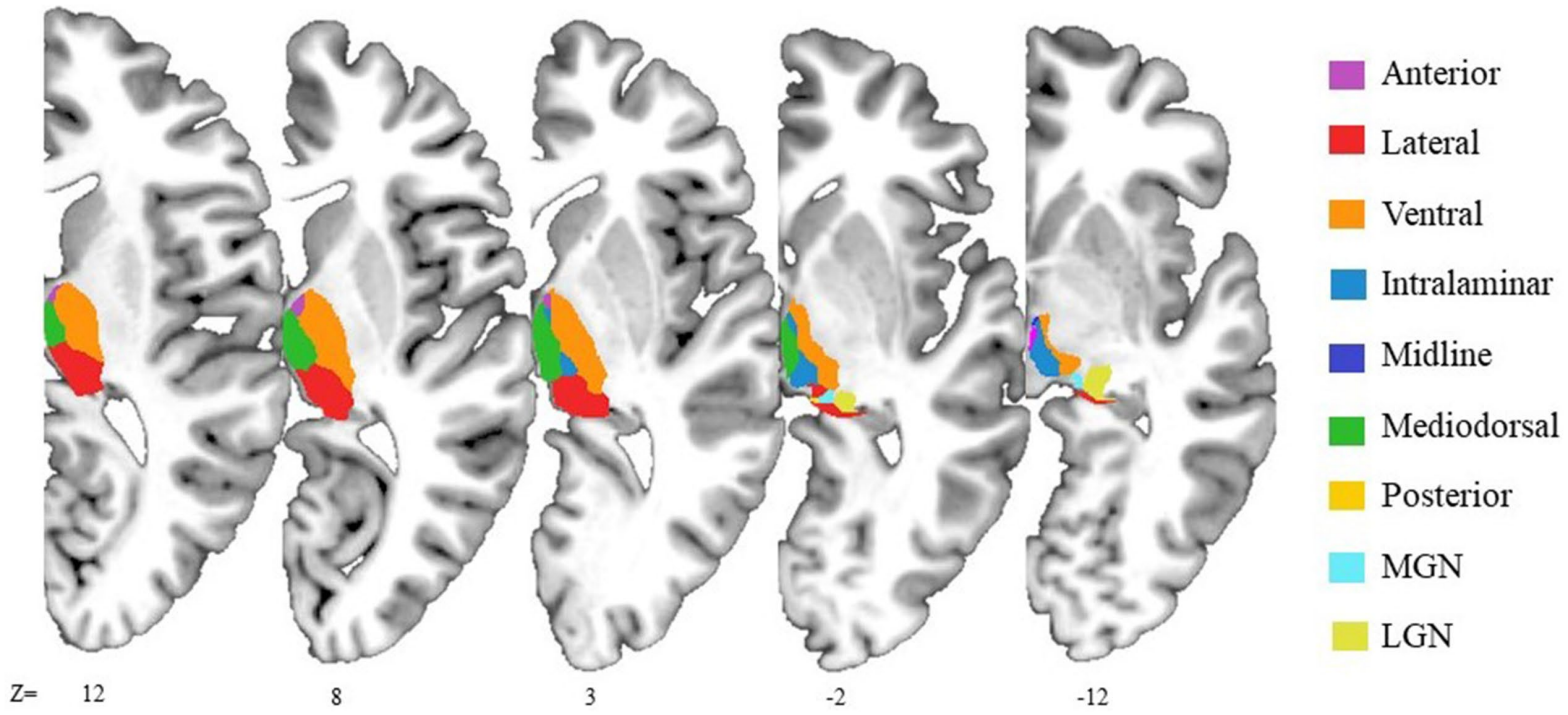
Illustrative representation of anatomical locations of the thalamic nuclear groups. The axial slices shown the thalamic segmentation generated by FreeSurfer on 3D T1-weighted image of the MNI305 template. The reticular nuclear group is not displayed due to its small size. It is adjacent to the ventral nuclear group, separated from the internal medullary lamina.

The following information has been extracted from each included study:

- The investigated thalamic nuclear group classified according to (15) that is based on previous studies (39–43); see Table 1 and Figure 1.
- The thalamic nucleus (if specified), classified according to (15). If an article used a nomenclature based on a different classification, we adapted it to the one specified in (15), i.e., in case of nuclei’s further division into parts depending on different criteria than (15), we considered the nucleus as a whole, and in case of nuclei not included in (15), we considered only the respective nuclear group (15). Importantly, for human studies reporting only the thalamic stereotactic coordinates in the Montreal Neurological Institute system, we extracted the anatomical label of regions according to the Automated Anatomical Labeling (44).
- The lateralization of each thalamic nucleus/nuclear group (if specified).
- The function of interest (i.e., consciousness, arousal, and wakefulness).
- The analyzed outcome measures (behavioral; instrumental).
- The sample characteristics, including species (humans; animals), the absence/presence of any pathology (healthy; pathological), and the sample size.
- The presence/absence of control conditions/groups.

**Table 1:** The table shows the thalamic nuclei classification adopted in the present work according to Nieuwenhuys et al. (15).

Nuclear group	Nucleus	Further divisions
Anterior	Anterodorsal (Ad)	
	Anteromedial (Am)	
	Anteroventral (Av)	
Intralaminar	Central Lateral (CL)	
	Central Medial (CeM)	
	Centromedian-Parafascicular (CM-Pf)	
	Paracentral (Pc)	
Lateral	Lateral Posterior (LP)	
	Laterodorsal (LD)	
	Pulvinar (PUL)	
		Anterior part (PULa)
		Inferior part (PULi)
		Lateral part (PULl)
		Medial part (PULm)
Lateral geniculate body	Dorsal (LGd)	
	Ventral (LGv)	
Medial geniculate body	Dorsal (MGd)	
	Medial (MGm)	
	Ventral (MGv)	
Mediodorsal	Mediodorsal (MD)	Magnocellular part (MDmc)
		Paralaminar part (MDpl)
		Parvocellular part (MDpc)
Midline	Paratenial (Pt)	
	Paraventricular (Pv)	
	Reuniens (Re)	
Posterior	Limitans (Li)	
	Posterior (Po)	
	Suprageniculate (Sg)	
Reticular	Reticular (TRN)	
Ventral	Ventral Anterior (VA)	Magnocellular division (VAmc)
	Ventral Lateral (VL)	Anterior part (VLa)
		Medial part (VLm)
		Posterior part (VLp)
	Ventral Posterior complex (VP)	Ventral Posterior Inferior (VPI)
		Ventral Posterolateral (VPL)
		Ventral Posteromedial (VPM)
		Ventromedial Posterior (VMpo)

The first column lists the thalamic nuclear groups, the second column lists the thalamic nuclei for each specific nuclear group; in the third column, further divisions are listed for each thalamic nucleus.

Then, for each thalamic nucleus/nuclear group, we recorded whether the evidence of the relationship with the function of interest contained in the study was either positive or negative. Consequently, a single study could contain a different number of positive/negative pieces of evidence depending on the number of considered thalamic nuclei/nuclear groups. This allowed us to count how many positive/negative pieces of evidence were retrieved for each thalamic nucleus/nuclear group.

### 2.4 Identification of thalamic nuclei most associated with the consciousness, arousal, and wakefulness

To identify the nuclei most associated with the function of interest, we first searched for the thalamic nuclear groups most associated with it by computing a numerical index. Specifically, we calculated the proportion of the number of positive pieces of evidence for each thalamic nuclear group (Pos_group_) over the total number of pieces of evidence for each thalamic nuclear group (Tot_group_) weighted for the natural logarithm (ln) of the Tot_group_, according to the following formulae: $\text{index}=\frac{{\mathrm{Pos}}_{\text{group}}}{{\mathrm{Tot}}_{\text{group}}}*\ln({\mathrm{Tot}}_{\text{group}})$ . Subsequently, following the same approach, we further checked whether within the thalamic nuclear group with the highest numerical index there were specific nuclei that could be considered pivotal for the function of interest. We thus computed a numerical index for each nucleus according to the following formulae: $\text{index}=\frac{{\mathrm{Pos}}_{\text{nucleus}}}{{\mathrm{Tot}}_{\text{nucleus}}}*\ln({\mathrm{Tot}}_{\text{nucleus}})$ where Tot_nucleus_ represents the total number of pieces of evidence for each nucleus and Pos_nucleus_ represents the total number of positive pieces of evidence for each nucleus.

## 3 Results

### 3.1 Literature search results

**Figure 2:**
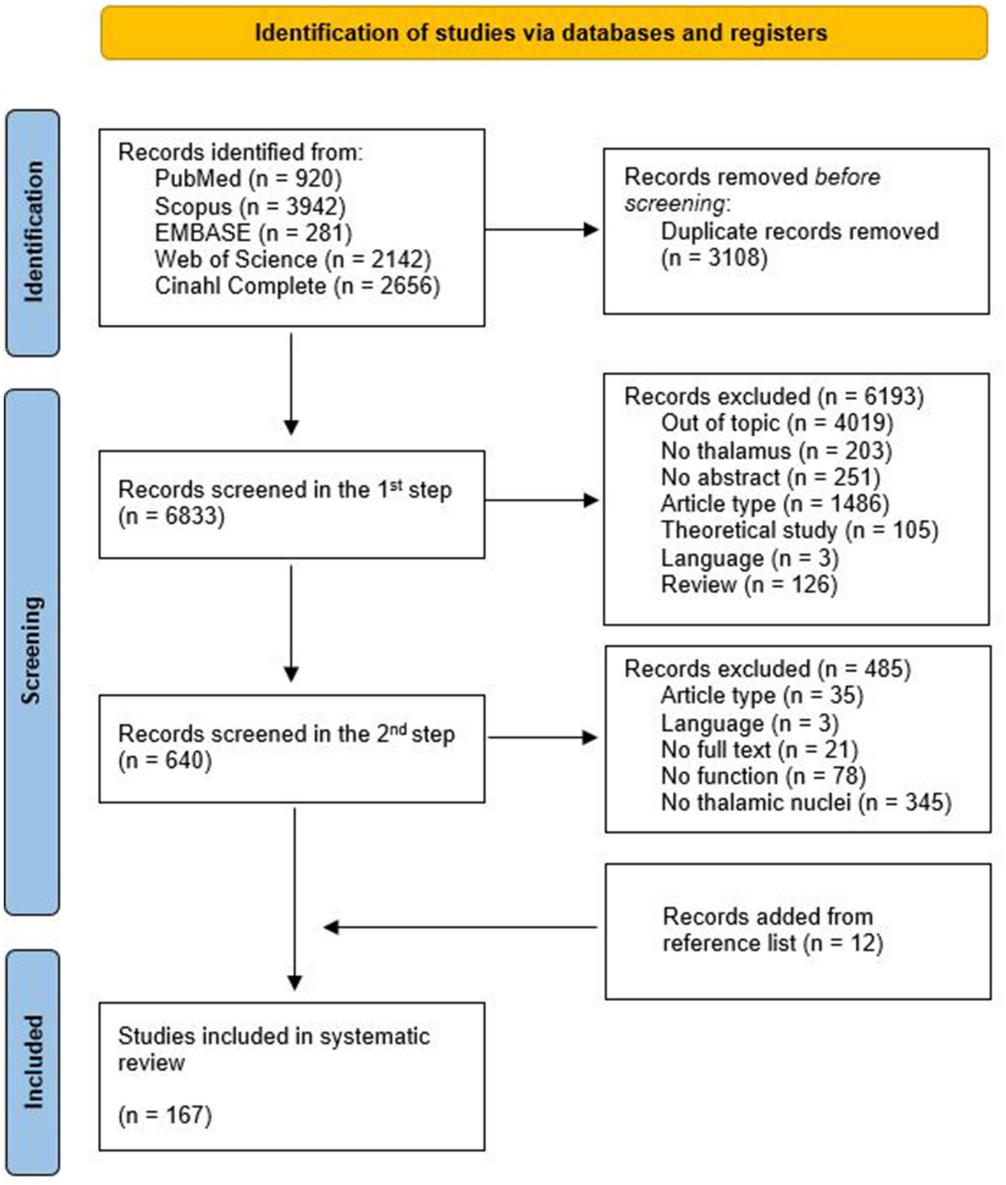
PRISMA flow diagram of this systematic review.

A total of 9,941 articles were retrieved (see Figure 2 for details). The automatic removal of duplicates resulted in 6833 articles.

During the first step, raters #1 and #2 agreed upon including 473 articles, whereas 167 were included by rater #3 who screened 424 records for which there was no agreement between raters. The Cohen’s Kappa value for inter-rater agreement in the first step was 0.65, implying ‘Good’ concordance between the raters (38). A total of 640 records were selected for full-text review.

During the second step, raters #1 and #2 agreed upon including 116 articles, whereas 39 were included by rater #3. The Cohen’s Kappa value for inter-rater agreement in the second step was 0.68, consisting in a ‘Good’ concordance between the raters (38).

After the second step, 155 articles met the inclusion criteria for this systematic review. Further 12 articles were identified by checking the references list of topic-relevant reviews. Consequently, a total of 167 articles were included in the systematic review.

Figure 2 illustrates the PRISMA flow chart of the articles’ selection process and the main reasons for exclusion.

### 3.2 Data extraction results

When considering the function of interest, most of the articles explored the relationship between a specific thalamic nucleus/nuclear group and consciousness (*n* = 137 out of 167), whilst a minority focused on arousal (*n* = 15 out of 167) and wakefulness (*n* = 15 out of 167). Moreover, 111 out of 167 articles related one of the above-mentioned functions to a single thalamic nucleus/nuclear group, whilst the remaining 56 articles considered at least two thalamic nuclei/nuclear groups. It is worth noticing that 21 out of 111 articles provided information only concerning the involvement of a specific nuclear group without any specification about the involvement of specific thalamic nuclei. Among them, 14 articles focused on the relationship between the function of interest and the intralaminar group, 5 considered only the anterior nuclear group, and the two remaining articles considered only the ventral group.

As for the hemispheric lateralization, most of the 167 included articles focused on specific thalamic nuclei/nuclear groups bilaterally (*n* = 76), many articles did not specify the lateralization (*n* = 52), whilst a minority focused on either left (*n* = 13) or right (*n* = 10) thalamic nuclei/nuclear groups. The remaining 16 articles heterogeneously considered left and right thalamic nuclei/nuclear groups across the sample.

Independently from both the function of interest and the considered thalamic nucleus/nuclear group, out of 167 articles, most of them (*n* = 95) explored their relationship by collecting only instrumental outcome measures being either neurophysiological (*n* = 50), imaging (*n* = 41), or both (*n* = 4). Sixty-one articles relied on both behavioral and instrumental outcome measures, whilst 11 articles explored the relationship between specific thalamic nuclei/nuclear groups and the function of interest by collecting only behavioral outcome measures.

A similar number of studies were conducted on animals (*n* = 81) or humans (*n* = 86). The former was mostly conducted on healthy animals (*n* = 67) during anesthesia (*n* = 45) or brain activity modulation adopting different techniques (*n* = 22). The articles on humans were mainly conducted on pathological populations (*n* = 76), mostly represented by patients with DOC after brain damage (*n* = 56). Importantly, a high proportion of studies conducted on human pathological populations (*n* = 22) was represented by single cases. Overall, many of the articles (*n* = 118) considered a control group/condition within the experimental design.

The 167 included articles contained a total of 346 pieces of evidence (see Supplementary Figure S1) of which 284 were positive, meaning that they supported the existence of a relationship between a specific thalamic nucleus/nuclear group and the function of interest.

### 3.3 Thalamic nuclei most associated to consciousness, arousal, and wakefulness

As mentioned above, since many articles reported data concerning thalamic nuclear groups, without focusing on specific nuclei, we first focused on the evidence by thalamic nuclear group. According to the computed numerical index, the intralaminar was the nuclear group most associated with the function of interest since it displayed the highest index followed by the ventral and mediodorsal ones (Table 2; see also Supplementary Tables S1a,b for the computation of human and animal evidence, separately).

**Table 2:** For each thalamic nuclear group, the table shows the total number of pieces of evidence (2nd column), number of positive pieces of evidence (3^rd^ column), and the numerical index used to identify the thalamic nuclear group most associated with the function of interest (4^th^ column; refer to the main text for the index computation).

Nuclear group	Tot_group_	Pos_group_	Index
Intralaminar	102	94	4.26
Mediodorsal	53	44	3.29
Ventral	77	58	3.27
Midline	27	22	2.68
Reticular	22	19	2.66
Lateral	32	23	2.49
Anterior	20	16	2.39
Posterior	6	5	1.49
Medial geniculate	3	2	0.73
Lateral geniculate	4	1	0.34

Considering the pieces of evidence specifying the thalamic nuclei within the intralaminar nuclear group, and following the same approach, we further checked for the specific intralaminar nuclei most associated with the function of interest. As a result, the CM-Pf was the nucleus most associated with the function of interest (Table 3; see also Supplementary Tables S1c,d for the computation of human and animal evidence, separately). It is worth noticing that we considered the CM-Pf complex instead of dividing the CM and Pf into two different nuclei since 15 pieces of evidence (45–59) considered the CM-Pf as a single thalamic nucleus. Thus, to avoid the removal of those results and given the anatomical and functional similarities between CM and Pf nuclei (60), we decided to consider the CM-Pf as a single thalamic nucleus.

**Table 3:** For each intralaminar nucleus, the table shows the total number of pieces of evidence (2nd column), number of positive pieces of evidence (3rd column), and the numerical index used to identify the intralaminar nucleus most associated with the function of interest (4th column; refer to the main text for the index computation).

Intralaminar nucleus	Tot_nucleus_	Pos_nucleus_	Index
Centromedian-parafascicular complex	40	38	3.5
Central lateral	25	23	2.96
Central medial	15	14	2.52
Paracentral	4	4	1.38

### 3.4 Summary of positive and negative evidence

We here provide an overview of the evidence retrieved for each nuclear group and their nuclei.

#### 3.4.1 Intralaminar nuclear group

Supplementary Table S2 lists the pieces of evidence about the relationship between the function of interest and the intralaminar nuclear group derived from 81 articles.

Much of the evidence from studies not reporting specific intralaminar nuclei focused on the structural (SC) and functional connectivity (FC) in brain-damaged patients. Indeed, the level of consciousness in these patients was associated to the degree of injury affecting the SC of the intralaminar-cortex (61–67) which, in turn, predicted consciousness recovery (67–71). This evidence is in line with the suggestion of the lesion of the ARAS comprising the intralaminar nuclei as a plausible pathogenic mechanism of impaired consciousness (72). When exploring FC between intralaminar, Default Mode Network (DMN), and posterior cingulate cortex during anesthesia-induced unconsciousness in healthy individuals, it decreased during the unconsciousness and was restored during the recovery phase (73). Further support was derived from evidence highlighting behavioral improvement after intralaminar electrical stimulation in DOC patients, which also determined significant improvement in cortical metabolism (74, 75).

By contrast, three pieces of evidence suggested the lack of contribution by the intralaminar nuclear group to anesthesia-induced unconsciousness in mice (76) and stimulation-induced arousal (77) in rats, as well as a non-significant association between intralaminar-frontal FC and recovery of consciousness in DOC patients (78).

##### 3.4.1.1 Centromedian-parafascicular complex (CM-pf)

A high amount of evidence was derived from studies adopting electrical stimulation targeting CM-Pf to boost the level of consciousness in DOC patients. Specifically, at the behavioral level, CM-Pf DBS resulted in arousal improvement during stimulation (49, 53) and the enhancement of the level of consciousness after the stimulation (47–50, 53, 54, 56, 79), determining changes in visual and motor abilities (55). At the neuronal level, electrical stimulation of the CM-Pf produced cortical arousal by increasing neuronal variability in all frequency bands (55), thus strengthening functional integrity and brain communication in DOC patients (46, 54, 55). The same results were observed by adopting CM-Pf electrical stimulation in mice (79) and non-human primates (51, 57, 80). Similarly, CM-Pf DBS in animal models of loss of consciousness resulted in an awake-like cortical state (i.e., increased spiking rate and decreased slow-frequency power and synchronization), enhanced FC with sensorimotor regions, and restored behavioral signs of consciousness (51, 81–83). Moreover, CM-Pf stimulation was associated with improved seizures’ outcome (84–88), as indicated by the reduction and even abolishment of both generalized tonic–clonic and convulsive seizures (84, 85) and atypical absences (85–87).

The CM-Pf role for consciousness was further supported by studies exploring the association between both structural and functional integrity and impaired consciousness in brain-damaged patients. Indeed, thalamic infarct and bilateral lesions involving the CM-Pf were frequently related to consciousness disorders (89–91). Specifically, DOC patients showed a neuronal loss (45, 92) and a significant reduction of metabolism in the CM-Pf (59), as well as a structural disconnection between the CM-Pf and both brainstem arousal nuclei (52) and cortical areas (93–95). These results on brain-damaged patients are in line with evidence derived from both animal and human studies adopting anesthesia to manipulate consciousness. Indeed, propofol suppressed the consciousness-related excitatory postsynaptic currents of the CM-Pf in a dose-dependent manner in rats (96). Similarly, Sukhotinsky et al. (97) reported CM-Pf involvement in both loss of consciousness and electroencephalogram (EEG) synchronization during anesthesia-induced unconsciousness after the microinjection of pentobarbital into the mesopontine tegmentum area in rats. Moreover, human studies on both healthy (98, 99) and pathological (33) populations reported the modulation of CM-Pf FC during the anesthesia-induced unconsciousness, which was gradually restored during the recovery phase (98, 99). Importantly, although cortical and subcortical FC of thalamic nuclei was progressively restored during the recovery phase, the CM-Pf is the only nucleus that fully restored FC to the ARAS 1 h after the emergence (98).

Finally, the remaining pieces of evidence deriving from electrophysiological studies on healthy individuals demonstrated the CM-Pf pivotal role in the modulation of behavioral arousal state during (58, 100) sleep/wake cycle (58) and sleep/arousal transition (58, 100).

Despite this large amount of positive evidence, a couple of negative pieces of evidence on stroke and epileptic patients was found (101, 102). Specifically, Hindman et al. (101) showed that the CM-Pf lesion is not sufficient to cause an impairment of consciousness in stroke patients, and Valentín et al. (102) found that CM-Pf DBS led to the remission of refractory status epilepticus without affecting the level of consciousness (102).

Considering the above-mentioned results altogether, CM-Pf surely plays a role for consciousness modulation due to its widespread cortical and sub-cortical connections allowing it to exert a significant influence over the whole brain activity deemed necessary to sustain the conscious state.

##### 3.4.1.2 Central lateral (CL)

Most of the evidence focusing on the CL was derived from animal studies adopting stimulation paradigms. Although with fewer pieces of evidence than CM-Pf, CL electrical stimulation increased physiological arousal and determined behavioral changes (e.g., motor activity improvement; increase of the level of consciousness) in awake mice (79), and both awake and anesthetized rats (57, 81, 103) and macaques (83, 104). Similarly, a study on an epileptic rat model described the increase of postictal cortical physiological arousal due to bilateral CL stimulation preventing slow activity and restoring behavioral responses (105). Moreover, a study stimulating deep cortical layer of awake mice suggested that cortico-CL interaction, along with other thalamic nuclei, drove long-lasting evoked EEG signals, and it was suppressed during anesthesia-induced unconsciousness (106). Furthermore, CL stimulation can bidirectionally influence consciousness. Indeed, CL electrical stimulation in an awake primate produced periods of perturbed consciousness in a frequency-dependent manner, characterized by similar features and involvement of the same networks that are pivotal in absence seizures (107). Similarly, optogenetic high-frequency stimulation of CL produced a whole brain activation resulting in increased behavioral arousal, while low-frequency stimulation led to behavioral arrest (108). However, it is important keeping in mind that in some of the above-cited studies the stimulation site spanned other thalamic nuclei in addition to the CL (79, 81, 108).

As for studies on humans, bilateral CL-DBS in a DOC patient modulated his behavioral responsiveness (48), whereas its discontinuation was associated with a significant responsiveness reduction (109). In line with these results, thalamic infarcts and bilateral lesions involving CL were associated with consciousness impairment (89–91) and, consistently, DOC patients showed neuronal loss in CL (45, 92) as well as symmetric structural disconnection between CL and the brainstem arousal nuclei (52). Similarly, decreased CL activity was detected during focal temporal (110) and limbic seizures (111) with loss of consciousness.

Evidence deriving from anesthesia-induced unconsciousness also showed the association between CL alpha oscillations and different behavioral states, suggesting the CL alpha coherence is one of the primary features of propofol-induced unconsciousness (112), in line with evidence showing the lack of preferential connections between CL and posterior/anterior cortical networks affected by anesthesia (33). Finally, evidence also showed its involvement in the SC pathway underlying the EEG synchronization and reversible loss of consciousness due to anesthesia through microinjecting pentobarbital into the mesopontine tegmentum area of rats (97).

Only two pieces of negative evidence were found for CL, both deriving from studies focused on epilepsy. Specifically, Kundishora et al. (113) showed that CL-DBS alone was not sufficient to restore an awake-like cortical state and González et al. (114) found no association between CL resting-state FC and the frequency of focal impaired consciousness seizures (114).

In summary, like what has been already seen for CM-Pf, CL activity can determine changes at both cortical and subcortical levels due to its bidirectional connections that possibly influence the level of consciousness and arousal.

##### 3.4.1.3 Central medial (CeM)

The evidence on CeM comes mainly from animal studies. Only three pieces of evidence were derived from the human population, showing a neuronal loss in the CeM of DOC patients (92), CeM involvement in the alpha network affected under anesthesia in epileptic patients (33), and CeM decreased metabolic activity during anesthesia-induced unconsciousness in healthy individuals (115).

Among animal studies, most of the evidence was derived from the adoption of anesthesia, suggesting the CeM is a brain site involved in the modulation of consciousness during anesthesia and a key hub in the pathway mediating recovery which can regulate prefrontal cortex oscillations (116). Specifically, although Fu et al. (117) reported CeM involvement only during the recovery from propofol anesthesia, Baker et al. (118) described CeM as a hub initiating the transition towards propofol-induced loss of consciousness, consistent with the results of the study by Muheyati et al. (116) showing a decrease of the loss of righting reflex duration in anesthetized rats after CeM-induced chemical lesion. Moreover, during the transition phase, changes in high-frequency oscillations (20–40 Hz) occurred first in the CeM and then in the cortex (117). Coherently, anesthetic infusion into the central thalamus (including the CeM) slightly reduced the cortical arousal induced by pedunculopontine tegmentum stimulation (119). Molecular studies also supported CeM involvement in consciousness modulation under anesthesia, demonstrating that CeM potassium channels inhibition is sufficient to restore consciousness in anesthetized rats (120, 121) and (122) the inhibition of the mitochondrial protein in the CeM caused hypersensitivity to anesthetics (122).

The remaining pieces of evidence were derived from studies adopting CeM stimulation paradigms (79, 123, 124). Specifically, bilateral DBS targeting the central thalamus including CeM increased both behavioral and physiological arousal, as demonstrated by increased motor activity and increased alpha, beta, and gamma waves (79). Moreover, this activation was time-blocked to the stimulation that, when ceased, determined the rats returning to an anesthetic status (123). Similarly, CeM-focused ultrasound stimulation in mice increased behavioral arousal, as demonstrated by increased locomotor activity (125). Consistently, optogenetic tonic activation of CeM neurons reliably induced rapid awakening from NREM whereas, optogenetic burst-like activation contributed to the initiation of cortical UP-states in the cingulate cortex that were synchronized over brain-wide cortical circuits through a relay in the anterodorsal (Ad) nucleus (124).

A single article provided negative evidence highlighting the involvement of other intralaminar nuclei than CeM underlying the EEG synchronization and reversible loss of consciousness induced by microinjection of pentobarbital into mesopontine tegmentum area in rats (97).

In summary, the CeM role in consciousness is mainly inferred from its importance as a target of anesthetic drugs and its connections with the anterior cortical areas.

##### 3.4.1.4 Paracentral (pc)

The studies supporting the role of Pc in consciousness and arousal overlapped some of the above-mentioned ones taking into consideration also other intralaminar nuclei. Indeed, evidence deriving from DOC patients showed a neuronal loss in Pc (92), whereas evidence deriving from anesthesia-induced unconsciousness in rats supported Pc involvement in modulating the EEG synchronization and reversible loss of consciousness (97). Moreover, electrical (79) and optogenetic (108) stimulation targeting the Pc increased behavioral and physiological arousal in mice and rats, respectively.

Overall, since Pc involvement in arousal and level of consciousness modulation was always accompanied by other intralaminar nuclei, it is possible hypothesizing that Pc carries-out a complementary, rather than pivotal, role for consciousness modulation.

#### 3.4.2 Mediodorsal nuclear group (MD)

The evidence supporting MD role for consciousness, wakefulness, and arousal was derived from 51 studies mainly conducted on humans (see Supplementary Table S3).

Specifically, most of the evidence is derived from studies on DOC patients showing thalamus-related structural abnormalities primarily located in the MD, including significant atrophy (34, 126), neuronal loss (involving both the magnocellular and parvocellular parts of MD) (52, 92, 126–130), and a lower number of spontaneous active units in the paralaminar MD (45). Consistently, paramedian infarcts involving MD were more frequently related to consciousness impairment (89–91, 131). Moreover, these MD structural and functional features were useful in discriminating the level of consciousness and long-term outcomes in DOC patients (34, 64, 126, 132–134), especially when thalamo-cortical connections were considered (64, 132, 135). For instance, thalamic tracks connecting MD to cortical areas were one of the main factors in distinguishing across different diagnostic categories (64) and clinical outcomes (132–134) of DOC patients. Moreover, when considering structural features, the MD total volume and its atrophy were predictors for consciousness recovery (34), and its total volume was negatively correlated to the disability level of DOC patients (126). Consistently, DOC was characterized by MD bilateral hyperintensity which returned to normal levels when consciousness recovered in two patients suffering from thiamine deficiency due to Wernicke’s encephalopathy (136, 137).

Furthermore, the role of the MD was supported by several studies on anesthesia-induced unconsciousness in both humans and animals. Specifically, the alpha coherence of MD, as well as CL and other sensory-motor nuclei, characterized the anesthesia-induced unconsciousness and oscillated in a “boot-up sequence” depending on behavioral states from induction to emergence (112). Similarly, Choi et al. (138) highlighted that MD cortical rhythms and their functional coupling are largely, but not exclusively, responsible for unconsciousness. Moreover, Ramadasan-Nair et al. (122) suggested the MD role also in determining anesthetic sensitivity for loss of consciousness: the inhibition of the mitochondrial protein in the MD caused hypersensitivity to anesthetics in mice. Consistently, anesthesia-induced unconsciousness modulated the MD regional activity (139–141) and its FC (33, 73, 99, 142, 143) in a dose-dependent manner (143), by suppressing its cortical connectivity during deep sedation which returned to the baseline during the recovery period (73). Importantly, evidence of MD parvo and magnocellular parts’ global signal co-activation coherently with arousal modulation in healthy individuals was found (141, 144). However, whilst CM-Pf-cortical FC was severely suppressed under anesthesia, MD-cortical connectivity was only moderately affected (99). Similarly, lidocaine infusion into the MD only slightly reduced the cortical activation induced by the pedunculopontine tegmentum stimulation in anesthetized rats (119).

The evidence deriving from studies on MD electrical stimulation was consistent in suggesting its role for consciousness. Specifically, when bilaterally stimulated in non-human primates, an awake-like cortical state was produced by increasing the spiking rate, decreasing slow-frequency power and synchronization, and reinstating higher-frequency power (57, 81), as well as behavioral arousal and motor activity in mice (79). Similarly, bilateral DBS of the central thalamus including paralaminar MD along with CL and CM-Pf modulated the behavioral responsiveness in a chronic DOC patient, improving both his cognitively-mediated and motor behaviors (48). Moreover, a recent study highlighted the MD role for the thalamo-cortical interactions underlying the physiological arousal elicited by cortical deep layer stimulation in mice, as well as in modulating perturbational complexity across different behavioral states (i.e., wakefulness and anesthesia-induced unconsciousness) (106). Furthermore, evidence on MD electrical stimulation (145) reported a decreased waking percentage and an increased slow wave sleep in MD neurotoxic-damaged rats, whilst MD stimulation through excitatory neurotransmitters produced a significant increase in total wake time (145). However, contrasting evidence reported a lack of MD neuron spike rate modulation across sleep/wake cycle, which was instead observed for the CeM (124), as well as a lack of activity of an important regulator of wakefulness (i.e., Neuropeptide S; NPS^+^) during the sleep/wake transition (146).

Evidence also support MD involvement within the network underlying the loss of consciousness during seizures (147–149): only rhythmic bursts of 30- to 40-Hz gamma activity of the MD characterized unconsciousness during seizures when compared to other thalamic nuclei (147). Consistently, Kundu et al. (87) reported a resolution of focal impaired awareness seizures in a patient implanted with the Responsive Neurostimulation System in the anterior nuclear group and CM-Pf, spanning the adjacent MD.

Despite the large number of positive pieces of evidence, negative evidence was found. For instance, when exploring the thalamic nuclei temporal dynamic activity underlying arousal state transitions in healthy individuals through fast functional Magnetic Resonance Imaging (fMRI), the MD was not among the thalamic nuclei activating first (100). Moreover, electrical stimulation of the MD in anesthetized epileptic rats did not induce slow neocortical activity (150). Similarly, the electrical stimulation of the prefrontal cortex via MD was insufficient to restore consciousness in anesthetized macaques (83), and MD chemical lesions did not affect the anesthesia-induced unconsciousness in rats (116). Finally, two studies reported a lack of restored FC between the MD and ARAS 1 h after the emergence from anesthesia-induced unconsciousness in healthy individuals (98), as well as an absence of relation between the MD-cortical pathways and unfavorable (i.e., death or DOC) long-term outcome in traumatic brain-injured patients (95).

In summary, the MD role for consciousness is mainly inferred from structural and functional data deriving from studies on DOC patients and its activity modulation under anesthesia-induced unconsciousness. However, it should be noted that in most of MD electrical stimulation studies, the stimulation sites spanned other thalamic nuclei.

#### 3.4.3 Ventral nuclear group

Supplementary Table S4 lists the pieces of evidence about the relationship between the function of interest and specific ventral thalamic nuclei derived from 52 articles. It is worth noticing that 3 studies (58, 151, 152) did not adopt the thalamic nuclei categorization considered in the present work, supporting the role of the ventral intermediate nucleus for arousal, wakefulness, and consciousness. Specifically, bilateral DBS affected the total sleep time and the sleep efficiency (151) and there was a gamma activity difference between sleep and wakefulness when recording local field potentials targeting the ventral intermediate nucleus in patients undergoing surgical DBS implantation (58). Moreover, the loss of coherence activity with the cortex during propofol anesthesia supported its role in consciousness too (152). Overall, the studies providing evidence for ventral intermediate nucleus’ role were a minority, whilst the most focused on the ventral posterior complex (VP).

##### 3.4.3.1 Ventral posterior complex (VP)

Only a few studies most conducted on brain-damaged populations and anesthesia-induced unconsciousness considered the VP as a whole, providing a quite similar amount of positive and negative pieces of evidence. Despite neuronal loss in VP being associated with Unresponsive Wakefulness Syndrome (UWS) (127, 129), and the SC from VP to cortex discriminating across patients with favorable and unfavorable 1-year outcomes (95), the evidence from the study by Maxwell et al. (92) showed no difference between UWS patients and severely disabled patients without DOC in the VP neuronal loss. Moreover, VP did not play a role in FC change during anesthesia-induced unconsciousness (99, 153) nor in influencing cortical activation underlying sleep/wake activity and the transition from wakefulness to sleep in mice (124). Nevertheless, a study described VP involvement in propofol-induced unconsciousness that was however determined by the inhibitory activity of the TRN over the VP (22).

On the contrary, a larger amount of positive than negative pieces of evidence was found for ventral posterolateral (VPL) and ventral posteromedial (VPM) nuclei. Although the single-cell recordings in the VPL, VPM, medial geniculate body, reticular formation, and cortex during wakefulness and anesthesia in cats failed to highlight which of these sites was the main responsible for anesthesia-induced unconsciousness, the authors pointed towards the involvement of both VPL and VPM in consciousness (154). Indeed, a study showed the VPL and VPM involvement in the thalamo-cortical interactions underlying the physiological arousal elicited by cortical deep layer stimulation in mice, as well as in the modulation of the perturbational complexity across behavioral states (i.e., awake and anesthesia) (106).

When looking at studies considering VPL, its SC was correlated with both the level of consciousness (93, 134) and the chance of recovery from DOC (93). Moreover, the FC between VPL and cortical areas significantly changed during anesthesia-induced unconsciousness (33, 155), as well as after cardiac arrest and during recovery in an ischemic rat model (156). When electrically stimulated during anesthesia-induced unconsciousness, VPL provoked neurophysiological changes like what has been recorded during wakefulness in macaques (81), and it is involved in the widespread networks underlying human absence seizures (86, 149). Furthermore, a few pieces of evidence also suggested VPL role in regulating arousal levels (100, 141) since it showed global signal co-activation coherently with arousal modulation (141) and it led, together with CM-Pf, the rest of the thalamus in determining arousal level changes as measured with fast fMRI in healthy individuals (100).

Similarly, the role of VPM has been explored by studies adopting anesthesia to induce unconsciousness in animals. Specifically, anesthesia-induced unconsciousness in rats were associated with VPM firing rate modulation (157, 158), VPM fast rhythms alteration, and an increase in thalamo-cortical coherence (159). Moreover, anesthesia determined a significant change in FC between VPM and cortex (160) which discharged coherently depending on the anesthetic concentration that determined, in turn, the level of unconsciousness in mice (161). Supporting this finding, the knockdown of the neuron-specific K-Cl co-transporter KCC2 in the VPM *in vivo* reduced the effect of anesthesia, whilst preventing KCC2 downregulation delayed the emergence time (158). Furthermore, the gamma/high gamma power of the VPM was associated with the level of consciousness (162, 163) and behavioral signs of arousal (162) in anesthetized rats. The VPM role was also supported by evidence of its involvement during seizures causing consciousness impairment both in humans (149) and rats (111, 164), and a further study showed a FC reduction from VPM to somatosensory cortex during sleep as compared to wakefulness in mice (165).

Of course, negative evidence for both VPL and VPM exist as well. Indeed, the atrophy degree of both VPL and VPM was not a significant predictor of brain-damaged patients’ 6-month recovery of consciousness (34). Moreover, VPM did not play a pivotal role in determining loss and recovery of consciousness after propofol anesthesia in rats as compared to TRN which was instead involved in inter-regional communication disruption with frontal areas (166). Moreover, when optogenetically stimulated in anesthetized mice, the VPM did not determine behavioral arousal and significant changes in EEG patterns (167). Similarly, VPL activity remained coupled with the cortical one from awake to unconsciousness in isoflurane-anesthetized rats (168), and, if electrically stimulated, it did not produce wakefulness, differently from other nuclei (123). Importantly, the same study showed that during anesthesia-induced unconsciousness, the fMRI water apparent diffusion coefficient did not change in VPL, and, similarly, local field potential power recorded outside the scanner did not decrease during anesthesia-induced unconsciousness in VPL (123).

Taken together, the existing data for VP pointed towards a role of this nucleus for consciousness and arousal when focusing on its specific nuclei (VPL and VPM), mainly due to their influence on the thalamo-cortical dynamics.

##### 3.4.3.2 Ventral lateral (VL)

When considering VL, there was a greater amount of positive than negative pieces of evidence, mainly deriving from studies on brain-damaged populations and anesthesia-induced unconsciousness.

Indeed, VL impairment due to polar-paramedian thalamic infarction, involving other thalamic nuclei, led to severe DOC with a quite high probability (131), and the VL atrophy degree predicted the 6-month recovery of consciousness in severely brain-injured patients (34). Moreover, the SC of VL (involving VL posterior part (134)) both discriminated across different levels of consciousness (64, 134) and distinguished patients with favorable and unfavorable (i.e., long-lasting DOC) outcomes after 1 year from the acute event (95). Furthermore, left VL preserved metabolism has been described as a distinctive feature of DOC patients’ improvement after treatment with transcranial Direct Current Stimulation targeting the left dorsolateral prefrontal cortex (169).

The evidence deriving from studies on anesthesia-induced unconsciousness was consistent in suggesting a role of VL for consciousness. Indeed, VL metabolic change and activation was found to be coupled with both the primary motor cortex and supplementary motor area during anesthesia-induced unconsciousness in both humans (170) and animals (153, 161). Moreover, VL is part of the functional network involving the DMN which showed a disconnection during anesthesia-induced unconsciousness and re-connection during the recovery time (73), and similar modulation was described when considering also VL posterior and anterior parts (33). Finally, evidence also showed VL involvement in the SC pathway underlying the EEG synchronization and reversible loss of consciousness when microinjecting pentobarbital into the mesopontine tegmentum area of rats (97).

The VL role for consciousness has also been supported by evidence showing its involvement in generating absence seizures (149) that were significantly reduced when bipolar DBS was used targeting the CM-Pf and passing through both the VPL and the VL posterior part bilaterally (86). On the contrary, VL-DBS during deep anesthesia in primates did not contribute either to consciousness recovery (80, 82) or to changing FC with cortical areas (82). Similarly, other evidence supported the lack of relationship between VL and consciousness since no changes were detected in its connectivity after anesthesia-induced unconsciousness (99) and during recovery both in humans (98) and rats (120). Similar negative evidence from fMRI studies highlighted that VL, and both VL anterior and posterior parts, did not contribute to arousal changes in healthy individuals (100, 144).

In summary, VL contribution to consciousness was supported by studies adopting heterogeneous methodologies that, however, well agree on its importance due to its connections with brain anterior areas.

##### 3.4.3.3 Ventral anterior (VA)

The studies considering the role of VA relying on brain-damaged populations and anesthesia-induced unconsciousness mainly provided positive evidence. Specifically, when bilaterally impaired after polar-paramedian thalamic infarction, VA was more probably associated with severe DOC as compared to its unilateral impairment; importantly, the impairment always encompassed other thalamic nuclei (131). Moreover, different levels of consciousness in DOC patients were related to the difference in both thalamo-cortical SC (64) and FC (132) involving VA, and the SC between VA and several cortical regions also differed between DOC patients and healthy controls (130). The role of VA was also supported by evidence highlighting its importance in predicting the recovery of consciousness in DOC patients (34, 95, 171).

Furthermore, evidence deriving from studies on anesthesia-induced unconsciousness showed a disconnection within the functional network involving VA and connecting the thalamus and the posterior parts of the DMN (i.e., precuneus and posterior cingulate cortex) which was re-connected during the recovery periods, when comparing brain metabolism and resting activity across the two conditions (73). Similarly, VA metabolism covaried with both the primary motor cortex and supplementary motor area during anesthesia-induced unconsciousness (170), thus suggesting VA FC modulation during anesthesia-induced unconsciousness as confirmed by neurophysiological data (33). Finally, as reported for VPM and VPL, VA also plays a role within the thalamo-cortical interactions underlying the physiological arousal elicited by cortical deep layer stimulation in mice, as well as in the modulation of the perturbational complexity across different behavioral states (106).

One negative piece of evidence was found highlighting other thalamic nuclei (i.e., CM-Pf and VPL) rather than VA as responsible for arousal changes in healthy individuals assessed through fast fMRI (100).

In summary, VA seems to be involved, together with other thalamic nuclei, within the thalamo-cortical network underlying consciousness modulation, playing a complementary role.

#### 3.4.4 Midline nuclear group

Supplementary Table S5 lists the evidence derived from 25 articles supporting the presence/absence of a relationship between the function of interest and specific midline nuclei. Importantly, all but two studies (33, 73) were conducted on animals.

Two studies reported contrasting evidence by considering the midline group overall. Indeed, whilst the study by Akeju et al. (73) showed its involvement in the mechanisms underlying the anesthesia-induced unconsciousness in humans, due to a decrease in its connectivity with DMN, Sukhotinsky et al. (97) reported no contribution of the midline nuclear group to the EEG synchronization and loss of consciousness after anesthesia-like state induction in rats after microinjecting pentobarbital into mesopontine tegmentum area.

##### 3.4.4.1 Paraventricular (Pv)

Most evidence derived from studies adopting chemogenetic and optogenetic manipulations in animals to explore the Pv role in consciousness and wakefulness. Specifically, chemogenetic and optogenetic activation of the Pv glutamatergic neurons, astrocytes, as well as orexinergic terminals and locus coerulus tyrosine-hydroxylase projections to Pv during anesthesia-induced unconsciousness prolonged the induction time and shortened the emergence time; coherently, their inhibition reduced the induction time and delayed the recovery time (25, 172–178). Furthermore, at a molecular level, Wu et al. (178) highlighted the role of the sodium leak channel of glutamatergic, but not GABAergic, neurons of Pv in modulating sedative effects of general anesthesia through the regulation of Pv neuronal activity. However, a recent study showed that a chemical-induced lesion to Pv accelerated the recovery time in gabodaxol-induced unconscious rats but not in diazepam-induced unconscious rats (116). Similar results on recovery time have been found by Bu et al. (179) who, however, did not obtain any result on the induction time. Alike, after performing a controlled-cortical injury in mice to induce DOC, the activation of the Pv glutamatergic neurons reduced the duration of the loss of consciousness, whereas Pv inhibition increased it (180). Furthermore, the activation of both the Pv glutamatergic neurons and the paraventricular hypothalamic nucleus–Pv circuit during sleep increased the wake time and decreased the NREM sleep time, while their inhibition reduced the wake time and increased the NREM sleep time (25, 181). Similarly, Ren et al. (182) revealed that chemogenetic and optogenetic activation of Glutamic acid decarboxylase 2-positive neurons in the dorsal raphe nucleus decreased the wakefulness time through monosynaptic inhibitory connections with the Pv. By contrast, Gao et al. (183) observed a different pattern of modulation after chemogenetic activation of the Pv type II neurons, consisting of a reduction of wake time and an increase in NREM sleep time.

Finally, evidence supporting the Pv role in consciousness also derived from anesthesia-based studies. Specifically, Liu et al. (184) explored the mechanisms underlying propofol-induced unconsciousness in mice, thus revealing a hyperpolarization of Pv occurring due to the modulation of the inhibitory currents via GABA_A_ receptors. Similarly, an immunofluorescence study on mice revealed that in Pv (and in VPM) the KCC2 expression is consistently downregulated during anesthesia-induced unconsciousness (158). Moreover, Pv contributed to promoting arousal from deep pharmacologically induced coma, given its recruitment after the stimulation of the anterior portion of the nucleus gigantocellularis of the ARAS (77). Instead, heterogeneous findings resulted from the adoption of c-Fos expression to study Pv activity during anesthesia. Specifically, on the one hand, two studies highlighted Pv-suppressed activity during anesthesia-induced unconsciousness (179) and increased activity during and after the emergence (179, 185); on the other hand, another study revealed significant activation of the Pv during anesthesia-induced unconsciousness (76).

Only two studies providing negative evidence were found, showing the lack of contribution of Pv in controlling cortical active/UP state and sleep modulations (124), as well as in the sleep/wake transition (146).

In summary, the Pv is a brain site involved in the modulation of consciousness and wakefulness, representing a key hub in the pathway mediating anesthesia-induced unconsciousness and sleep/wake cycle.

##### 3.4.4.2 Reuniens (Re)

Only the study by Weiner et al. (33) provided evidence of Re involvement in promoting anesthesia-induced alpha network anteriorization to achieve loss of consciousness in humans. Contrarily, two studies provided evidence against Re involvement in arousal modulation, either after deep pharmacologically induced coma (77) or during NREM sleep (124) in animals.

##### 3.4.4.3 Paratenial (Pt)

A single study (186) supported Pt contribution to the loss of consciousness secondary to focal limbic seizures. Specifically, by electrically inducing focal limbic seizures in rats, the authors found a concurrent decreased activity in Pt and increased cortical slow wave activity during the ictal period, possibly due to the inhibition of the Pt excitatory output to the basal forebrain, thus contributing to reduced arousal from basal forebrain to the cortex.

#### 3.4.5 Reticular nucleus (TRN)

The evidence supporting TRN role for consciousness, wakefulness, and arousal was derived from 22 studies mainly conducted on animals while adopting either anesthesia to induce unconsciousness or brain stimulation techniques targeting TRN (see Supplementary Table S6).

Although a study highlighted increased intrinsic excitability of GABAergic TRN neurons during propofol anesthesia and a consequent inhibitory influence over the VP glutamatergic neurons (22), other studies described a reduction of the electrical synaptic strength of TRN GABAergic parvalbumin-expressing neurons (187), along with a modulation of the reticulo-thalamo-cortical communication in a dose-dependent manner, with greater effects at deep levels of anesthesia (160, 166, 188). Moreover, anterior TRN optogenetic and chemogenetic activation shortened the emergency time from propofol-induced unconsciousness (189) and affected the number of transitions between wake and NREM sleep (20), whereas its inhibition delayed the recovery time from propofol-induced unconsciousness (189). The study conducted by Herrera et al. (190) went in the same direction since optogenetic activation of the GABA neurons of the lateral Hypothalamus-TRN circuit induced a rapid arousal during NREM sleep. Similarly, optogenetic activation of basal forebrain GABAergic terminals in the TRN also strongly promoted cortical activation and behavioral emergence from anesthesia (191). At a neurophysiological level, the optogenetic activation of the TRN induced a rapid increase of the frequency power in the delta band along with a decrease in the beta and gamma bands of the ipsilateral somatosensory cortex (192); at a behavioral level, it reduced the awake and increased the NREM sleep times (192). Contrary to the above-mentioned results, the study by Yi et al. (193) provided contrasting evidence on the role of the anterior TRN GABAergic neurons in the regulation of the mechanisms behind general anesthesia. Indeed, chemogenetic and optogenetic activation of the anterior TRN GABAergic neurons shortened the induction time of isoflurane anesthesia and delayed the recovery time from both propofol- and isoflurane-induced unconsciousness (193). Moreover, when electrical stimulation was applied to the cortical deep layer in mice, the TRN drove the physiological arousal elicited by the stimulation and modulated the perturbational complexity across different behavioral states (i.e., awake and anesthesia) (106).

Three studies provided positive evidence relying on results from brain-damaged population. Specifically, traumatic coma in humans was related to a disconnection of brainstem arousal nuclei from TRN (52), and the recovery from coma was associated with an increased FC between TRN and basal ganglia in rats (194). Similarly, the integrity of TRN projections to the frontal cortex was predictive of a one-year favorable outcome after severe traumatic brain injury in humans (95).

Consistent evidence for a relationship between TRN and consciousness derived from studies on epilepsy in animals. Indeed, the TRN showed an increased metabolism (164) and tonic firing neuronal activity (21) during absence seizures, associated with the formation of cortical spike-and-wave discharge. Furthermore, deletion of the Phospholipase C β1 in the TRN induced spike-and-wave discharges and reduced TRN excitability, thus causing absence seizures (195). Similarly, Gad1 dejection in TRN, a gene responsible for the synthesis of GABAergic neurotransmitters, caused spike–wave discharge in rats (196).

Despite the consistency of the positive pieces of evidence, there were also negative ones. Specifically, Mesbah-Oskui et al. (197) showed that the optical stimulation of the left TRN was not sufficient to maintain the anesthesia-induced unconsciousness. Moreover, TRN did not contribute to the cortical arousal elicited by the pedunculopontine tegmentum stimulation (119) nor to the EEG synchronization and loss of consciousness after anesthesia-like state induction (97).

Taken together, the results on TRN agree in supporting its importance for consciousness, mainly due to its inhibitory role over the other thalamic nuclei and considering that most of the studies were conducted on animals thus, allowing a more precise characterization of TRN neuronal mechanisms compared to what usually done in humans.

#### 3.4.6 Lateral nuclear group

None of the 22 retrieved studies considered the lateral nuclear group as a whole (see Supplementary Table S7).

##### 3.4.6.1 Pulvinar (PUL)

Studies on brain-damaged patients showed an association between impaired consciousness and both PUL FC and SC (52, 64, 93, 134, 198, 199). For instance, the PUL medial part was a functional connective node significantly related to the level of consciousness in DOC patients (134), and the strength of PUL SC was associated with the recovery from DOC (93, 134). Similarly, evidence from magnetic resonance spectroscopy in DOC patients showed that the ratio of the brain metabolites of PUL medial part (but not for the lateral one) was associated with a negative outcome at 12 months (199).

Similar findings were suggested by studies on epileptic patients. Indeed, the degree of the loss of consciousness in temporal lobe epilepsy was related to the involvement of PUL within the seizure network (148), and the electrical stimulation of the PUL medial part reduced the mean duration of the tonic phase and the severity of consciousness alteration (200). Similarly, Kundu et al. (87) reported a resolution of focal impaired awareness seizures in an epileptic patient implanted with a closed-loop Responsive Neurostimulation System targeting the anterior nuclear group and the CM-Pf, spanning the PUL.

Moreover, anesthesia-induced unconsciousness modulated PUL coherent alpha networks (33) and its global signal co-activation (141).

By contrast, unlike other thalamic nuclei, PUL was not relevant for changes in arousal state when considering resting-state fMRI (144), nor was one of the first thalamic nuclei to become active during arousal state transitions (100). Finally, neither its atrophy degree (34) nor the fiber density of the pulvinar-cortical tracks (95) in brain-damaged patients predicted the DOC patients’ outcome.

Taken together, the results about the role of PUL for consciousness are heterogeneous depending on which part of PUL is considered. Its role is particularly supported by data deriving from clinical populations being either DOC or epileptics patients.

##### 3.4.6.2 Lateral posterior (LP)

A similar amount of positive and negative pieces of evidence characterized the studies considering the LP role for consciousness.

When considering its FC (201) and SC (52) in DOC patients, there was a correlation with both the level of consciousness (201) and the outcome (52). Moreover, during anesthesia-induced unconsciousness, there was a disruption of the posterior alpha network structurally connected with the LP (33).

On the contrary, LP neuronal loss was neither predictive of the 6-month outcome (34) nor distinguished across different levels of consciousness (92, 127, 129) in brain-damaged patients.

In summary, the available data did not allow to conclude for a LP pivotal role for consciousness.

##### 3.4.6.3 Laterodorsal (LD)

Few studies considered the LD, all providing positive evidence for its involvement in consciousness.

Indeed, LD lesion was associated with the severity of DOC (34) and worst outcome at follow-up (34, 131). Consistently, Tenney et al. (164) reported LD involvement in the generation and maintenance of absence seizures by adopting fMRI. Moreover, propofol-induced alpha oscillations were associated with different behavioral states in healthy individuals (33) and rats (112). For instance, before the recovery from anesthesia-induced unconsciousness, alpha coherence between superficial cortical layers and LD (along with the MD and CL nuclei) recovered, consistent with a “boot-up sequence” during the emergence from anesthesia-induced unconsciousness (112).

Overall, besides the paucity of studies considering LD, it should be noted that LD involvement was accompanied by other thalamic nuclei, suggesting thus only a complementary LD role for consciousness.

#### 3.4.7 Anterior nuclear group

All but four of the 20 retrieved articles provided positive evidence for the anterior group role for consciousness, wakefulness, and arousal (see Supplementary Table S8).

When considering anesthesia-induced unconsciousness, the anterior nuclear group connectivity pathway was involved in the loss of consciousness at both structural and functional levels [i.e., connectivity with both posterior cingulate cortex (143) and mesopontine tegmentum area area (97)]. Moreover, a high number of studies adopted anterior nuclei-DBS in epileptic patients demonstrating that it increased vigilance and arousal both during sleep and wakefulness (202, 203), interrupted sleep (203), and was followed by the disappearance of tonic–clonic seizures and complex spikes and waves (204, 205). Similarly, the Responsive Neurostimulation System implanted in the anterior nuclear group, targeting also the CM-Pf and the adjacent MD and PUL, stopped focal impaired awareness seizures in a patient suffering from frontotemporal epilepsy (87). Importantly, Singh et al. (206) found enhanced synchrony in alpha and beta bands recorded with stereotactic EEG targeting the anterior nuclei during focal seizures with impaired awareness and focal to bilateral tonic–clonic seizures but not during focal aware seizures thus, supporting the role of anterior nuclei activity in determining changes at the level of consciousness. Finally, studies on brain-damaged patients showed structural and functional abnormalities of the anterior nuclear group, including atrophy (34, 131), fibers’ density decrease within the cortical pathway (52, 130), and altered metabolites ratio (199) relating both to acutely and long-lasting impaired consciousness.

On the contrary, two studies did not report an association between the anterior nuclear group and consciousness in brain-damaged patients (89, 95). Furthermore, Feng et al. (111) showed that, during epilepsy, the anterior nuclear group played a role in seizure propagation rather than in consciousness-related disturbances.

##### 3.4.7.1 Anteroventral nucleus (Av)

Only three studies considered the Av. Specifically, it belonged to the posterior alpha network connecting the frontal cortical regions and the higher-order sensory thalamic nuclei showing a decrease of coherence during propofol-induced unconsciousness (33). Moreover, a recent study suggested its role in the thalamo-cortical interactions underlying the physiological arousal elicited by cortical deep layer stimulation in mice (106). On the contrary, Av did not play a pivotal role during sleep/wake transition in healthy individuals assessed through fast fMRI (100).

Overall, despite the paucity of studies, it is possible hypothesizing that the Av carries-out a complementary role within the thalamo-cortical interaction underlying the consciousness modulation, rather than a pivotal role.

##### 3.4.7.2 Anterodorsal nucleus (Ad)

Only a study supporting the Ad role in propagating the cortical activation induced by CeM optogenetic activation was found. Specifically, CeM optogenetic stimulation during natural sleep initiated the UP states in the cingulate cortex that propagated in the visual cortex through the Ad activity (124), meaning that Ad acted as a relay.

#### 3.4.8 Posterior nuclear group

Despite the paucity of studies (*n* = 6) considering the role of the posterior nuclear group for consciousness, all but one provided positive evidence (see Supplementary Table S9).

When microinjecting pentobarbital into the mesopontine tegmentum area, the posterior nuclear group was involved in the SC pathway underlying the loss of consciousness in rats (97); similarly, alpha coherence of the posterior nuclear group, as well as other thalamic nuclei, characterized the anesthesia-induced unconsciousness and oscillated in a “boot-up sequence” from induction to emergence (112). Furthermore, it was involved, together with other thalamic nuclei, in the generation and maintenance of absence seizures as assessed with fMRI in rats (164). By contrast, a study highlighted the lack of contribution of the posterior nuclear group to the recovery of consciousness in anesthetized rats (120).

##### 3.4.8.1 Suprageniculates-limitans nuclei (Sg-Li)

Only the study by Weiner et al. (33) provided evidence of Sg-Li involvement in anesthesia-induced unconsciousness, reporting a disruption of the posterior alpha network structurally connecting Sg-Li to frontal cortical areas.

##### 3.4.8.2 Posterior nucleus (Po)

A single study was found, highlighting Po involvement in the thalamo-cortical interactions underlying the physiological arousal elicited by cortical deep layer stimulation in mice, as well as in modulating the perturbational complexity across behavioral states (106).

#### 3.4.9 Medial geniculate body

Three studies explored the relationship between the medial geniculate body and consciousness providing mixed evidence (see Supplementary Table S10).

Intracranial recordings in epileptic patients under anesthesia showed connectivity disruption between the posterior alpha network and association and sensory thalamic nuclei, including the medial geniculate body (33). Similarly, intracranial recordings in anesthetized cats detected the involvement of the medial geniculate body, as demonstrated by the depression of its neuronal firing during loss of consciousness, although the same pattern was described for other thalamic nuclei without determining which of them played a pivotal role (154). By contrast, when exploring the predictors for recovery of consciousness in DOC patients, its atrophy did not predict the 6-month outcome (34).

#### 3.4.10 Lateral geniculate body

Four studies considered the role of the lateral geniculate body for consciousness mainly providing negative evidence (see Supplementary Table S11).

The positive evidence consisted of what was already reported for the medial geniculate body, namely a connectivity loss during anesthesia-induced unconsciousness involving also the lateral geniculate body (33). Differently, when exploring the brain activity underlying arousal state transitions in healthy individuals through fast fMRI, the lateral geniculate body was not among the thalamic nuclei activating first (100). Similarly, it was not involved in the loss (52) and recovery of consciousness (34) in severely brain-injured patients. Indeed, the post-mortem tractography on a traumatic coma patient showed the partial sparing of connections between the lateral geniculate body and ARAS (52), and its atrophy degree did not predict the 6-month recovery of consciousness (34).

## 4 Discussion

The pivotal role of the thalamus for consciousness is well known within the underlying feedforward and feedback pathways. Nonetheless, literature still lacks an agreement on which thalamic nuclei are primarily involved in the generation, maintenance, and modulation of consciousness.

After having systematically reviewed all the studies published in the last 20 years exploring the relationship between thalamic nuclei/nuclear groups, we found a different number of pieces of evidence supporting the above-mentioned relationship across distinct thalamic nuclei/nuclear groups. For this reason, we first searched for the thalamic nuclear group most associated with consciousness which resulted in the intralaminar nuclear group, followed by the mediodorsal and ventral nuclei.

This evidence derives mainly from clinical human studies involving patients with DOC and absence epilepsy and adopting intralaminar DBS to modulate the level of consciousness. According to these results, the key role of the intralaminar nuclear group for consciousness is supported by its anatomo-functional features. Broadly speaking, when considering the microscopic level, thalamic cells can be divided into two classes: core neurons projecting to the middle cortical layers, and matrix neurons projecting to the superficial or deep cortical layers (207, 208). Although most of the thalamic nuclear groups generally contain a mix of core and matrix neurons, the intralaminar neurons are the only ones that exhibit both core- and matrix-like properties (209). These functional properties allow the intralaminar neurons to influence a wide range of cortical and subcortical areas (29), thus representing an ideal candidate for sustaining and modulating consciousness (29, 210). Taking in mind these anatomo-functional features, it is not surprising that, over the years, a growing number of studies investigating the NCCs have focused on intralaminar nuclei, as highlighted by the results of the present systematic review (see Supplementary Figure S2). Since the intralaminar nuclear group is composed of different nuclei, we further checked whether a specific intralaminar nucleus was most associated with consciousness resulting in the CM-Pf, followed by the CL.

The CM-Pf is relatively larger than other intralaminar nuclei (60, 211), and, together with CL, occupies the largest part of the intralaminar nuclear group. When considering its connectivity map, CM-Pf shows prominent connectivity with both subcortical areas, including the brainstem nuclei and the striatum, and motor and sensory cortical areas (29). The strong connections with the basal ganglia and their reciprocal connections with the cortical areas support the crucial role of CM-Pf in alerting and orienting responses to external stimuli, motor function, and, therefore, the modulation of arousal and consciousness level (60, 212). The results of the present systematic review showed that most of the evidence exploring the CM-Pf’s role within the NCCs comes from electrical stimulation studies on DOC patients producing significant changes in the level of consciousness. Indeed, a recent systematic review on the clinical effects of DBS (32) revealed the CM-Pf as the region most frequently targeted and with the best effective ratio for DOC patients, resulting in both an initial arousal increase and sustained improvement in the level of consciousness. A possible explanation for these effects has been postulated within the framework of the mesocircuit model (31, 213). Specifically, CM-Pf stimulation may relieve the inhibitory effect of the basal ganglia over the thalamus thus, restoring the normal function of the cortico-striatal-thalamus-cortical integration system in DOC patients, in whom brain damage reduced the background synaptic activities (31, 214). Moreover, CM-Pf role for consciousness could be further supported by data focusing on clinical disorders not considered in this systematic review and often associated with impaired awareness, such as schizophrenia (215, 216), hemispatial neglect (217–219) and somatoparaphrenia (218). For instance, a reduced CM-Pf volume has been observed in schizophrenic patients (215), as well as hemispatial neglect following thalamic infarct involving the CM-Pf (217). Consistently, a recent study highlighted the reduction of sensorimotor neglect in a rat model of Parkinson’s disease treated with CM-Pf-DBS (218). Although speculative, these data stress the need to broaden the study of NCCs to these clinical conditions.

Despite having a lower proportion of positive evidence, the CL nucleus seems to play a key role in the circuit for consciousness as well. Indeed, analogously to CM-Pf, CL electrical stimulation can produce an increase in physiological arousal, and thus an improvement in the level of consciousness. However, as opposed to CM-Pf, the evidence came mainly from animal studies. Taken together, the studies’ results suggest that both CL and CM-Pf contribute to the generation, maintenance, and modulation of consciousness, even though with different properties that remain to be clarified (210). Indeed, given the limited data available, to date, it is difficult to assess whether the positive effect during electrical stimulation is mainly driven by CM-Pf or CL. This is partly due to the anatomical proximity of these nuclei which, therefore, prevents to exclude a propagation of the electric current through the tissues during the DBS, and partly related to their simultaneous stimulation (especially in human studies) making it difficult to isolate the causality of the effect. However, by considering the anatomo-functional features of these two nuclei, there are substantial differences. Indeed, CL is characterized by matrix-style projections (207) mainly to the frontal and parietal cortices, and basal ganglia (29, 220–222), while CM-Pf is characterized by core-style projections to the basal ganglia, and matrix-style projections to cortical sensorimotor areas (29). One could hypothesize that, given the strong connections with the basal ganglia, CM-Pf dominates the effect on consciousness (31). However, a study adopting a more specific microstimulation approach suggested CL as the best candidate, reporting physiological arousal effects only when the stimulation site was centered on CL and not when limited to CM-Pf (83). In addition, these results have been confirmed by a computational model study (223) showing restoration of the wake-like flow of bidirectional information after the simulated stimulation of a site with a high proportion of matrix-to- core populations (i.e., the CL) rather than a site with a lower proportion of matrix-to- core nuclei (i.e., the CM-Pf). Taken together, these results emphasize the evidence for a pivotal role of the intralaminar nuclei in consciousness. However, future studies are needed to further frame the different mechanisms behind the modulation of consciousness played by the CM-Pf and CL.

Furthermore, when considering our results, it should be stressed that identifying the thalamic nuclei most related to consciousness can be useful from a therapeutic point of view. Indeed, it could help in choosing a precise and reliable target region of neuromodulation aiming at boosting the level of consciousness in DOC patients (e.g., DBS; low-intensity focused ultrasound; pharmacological treatment). Nevertheless, while this approach is practical in a clinical context, it must be emphasized that linking consciousness to a single nucleus may be overly reductionist, since current evidence highlights networks of subcortical and cortical areas underlying consciousness rather than a single driver nucleus. Indeed, over the years, the subcortical neuromodulatory systems’ activity has been considered a background condition for enabling consciousness and ensuring the adequate arousal state and afferent inputs to modulate and interconnect the cortical areas, including the fronto-parietal network (224) as well as temporal, parietal, and occipital areas (225) as a “posterior hot zone” for consciousness (226).

This systematic review shows how other nuclei could play a role in modulating consciousness, such as the MD and the ventral nuclei. Although MD is a nuclear group according to (15), it also represents a thalamic nucleus characterized by specific properties (45) and connections (227) and occupying a large anatomical thalamic portion as compared to the other thalamic nuclei (15). If we had considered MD as a thalamic nucleus instead of a nuclear group, its index would have been comparable to that of CM-Pf suggesting the relevance of both nuclei. Like what reported for the intralaminar nuclei, the evidence supporting MD role derives from studies highlighting a strict link between MD structural/functional damages and DOC. However, in most of the retrieved studies on DOC patients, thalamic nuclei other than MD have been considered showing a pivotal role for consciousness as well. In this line, for many of the considered stimulation studies the stimulation sites spanned multiple thalamic nuclei, even belonging to different nuclear groups, further supporting the contribution of several thalamic nuclei to consciousness rather than one. Despite the computed index identifying the intralaminar as the nuclear group most associated with consciousness, the methodological characteristics of the retrieved studies should also be considered when interpreting the evidence. The studies exploring the role of the intralaminar group for consciousness are single cases in a quite high percentage (around 22%), and a large proportion of studies did not have a control condition (around 45%), independently to provide positive or negative evidence. The studies exploring the role of other nuclear groups are characterized by larger samples and either a control condition or a control group with higher percentages. Moreover, the risk of publication bias should be considered, since negative findings are often underrepresented in the literature, potentially leading to an incomplete assessment of the evidence. For these reasons future investigations should better weigh the evidence considering both the nature of findings (i.e., positive and negative) and some methodological features, including the population (human or animal), the sample size, and the presence of a control condition. Furthermore, given the heterogeneity characterizing the nomenclature of thalamic nuclei, a standardization is needed to facilitate and guarantee a correct data comparison and the interpretation of results.

In conclusion, this work confirms the important role of intralaminar nuclei for consciousness, and, among them, the CM-Pf results the most positively associated with it (i.e., having the highest proportion of positive pieces of evidence), suggesting its importance as a possible target for neuromodulation. Of course, further studies with more rigorous methodology are needed to better clarify the mechanisms distinguishing the CM-Pf from the other intralaminar nuclei, such as CL for sustaining and modulating consciousness. Finally, it is important to stress the need for further studies better elucidating the role of non-intralaminar thalamic nuclei for consciousness as well, especially considering the bias in the recent literature in focusing on the intralaminar nuclei.

## Data Availability

The original contributions presented in the study are included in the article/Supplementary material, further inquiries can be directed to the corresponding author/s.
