# Supplementary material for: Thalamus and consciousness: a systematic review on thalamic nuclei associated with consciousness
Source: Front Neurol. 2025 Jun 18;16:1509668. doi: 10.3389/fneur.2025.1509668 (PMC12241866; doi:10.3389/fneur.2025.1509668)
Supplement: Supplementary file 1 [file Supplementary_file_1.docx]

Supplementary Material

# Supplementary Search Strategy

***MEDLINE/Pubmed (advanced search)***

#1 (search string related to the domain of thalamic nuclei)

((((((((((thalamus[MeSH Terms]) OR (thalamic nuclei[MeSH Terms])) OR (anterior thalamic nuclei[Title/Abstract])) OR (geniculate bodies[Title/Abstract])) OR (intralaminar thalamic nuclei[Title/Abstract])) OR (lateral thalamic nuclei[Title/Abstract])) OR (mediodorsal thalamic nucleus[Title/Abstract])) OR (posterior thalamic nuclei[Title/Abstract])) OR (ventral thalamic nuclei[Title/Abstract])) OR (centromedian nucleus thalamus[Title/Abstract])) OR (midline thalamic nuclei[Title/Abstract])

#2 (search string related to the domains of arousal/consciousness)

((((arousal[MeSH Terms])) OR (awareness[MeSH Terms])) OR(consciousness[MeSH Terms])) OR((wakefulness[Title/Abstract]))

#1 AND #2 AND "english"[Language]))

***SCOPUS (advanced search)***

#1

( TITLE-ABS-KEY ( thalamus ) OR TITLE-ABS-KEY ( thalamic AND nuclei ) OR TITLE-ABS-KEY ( anterior AND thalamic AND nuclei ) OR TITLE-ABS-KEY ( geniculate AND bodies ) OR TITLE-ABS-KEY ( intralaminar AND thalamic AND nuclei ) OR TITLE-ABS-KEY ( lateral AND thalamic AND nuclei ) OR TITLE-ABS-KEY ( mediodorsal AND thalamic AND nucleus ) OR TITLE-ABS-KEY ( posterior AND thalamic AND nuclei ) OR TITLE-ABS-KEY ( ventral AND thalamic AND nuclei ) OR TITLE-ABS-KEY ( centromedian AND nucleus AND thalamus ) OR TITLE-ABS-KEY ( midline AND thalamic AND nuclei ) OR TITLE-ABS-KEY ( intralaminar AND thalamic AND nuclei ) OR TITLE-ABS-KEY ( lateral AND thalamic AND nuclei ) OR TITLE-ABS-KEY ( mediodorsal AND thalamic AND nucleus ) OR TITLE-ABS-KEY ( posterior AND thalamic AND nuclei ) OR TITLE-ABS-KEY ( ventral AND thalamic AND nuclei ) )

# 2

(TITLE-ABS-KEY ( arousal ) OR TITLE-ABS-KEY ( awareness ) OR TITLE-ABS-KEY ( wakefulness ) OR TITLE-ABS-KEY ( consciousness ) )

#1 AND #2 AND ( LIMIT-TO ( LANGUAGE , "English" ) )

***EMBASE*** ***(advanced search)***

#1

('thalamus'/exp/mj OR 'thalamus nucleus'/exp/mj OR 'thalamus anterior nucleus':ti,ab,kw OR 'geniculate body':ti,ab,kw OR 'thalamus intralaminar nucleus':ti,ab,kw OR 'thalamus lateral nucleus':ti,ab,kw OR 'thalamus dorsomedial nucleus':ti,ab,kw OR 'thalamus posterior nucleus':ti,ab,kw OR 'thalamus ventral nucleus':ti,ab,kw OR 'thalamus medialis centralis nucleus':ti,ab,kw OR 'thalamus midline nucleus':ti,ab,kw)

# 2

('arousal'/exp/mj OR 'awareness'/exp/mj OR 'consciousness'/exp/mj OR wakefulness:ti,ab,kw)

#1 AND #2 AND english:la

***Web of Science*** ***(advanced search)***

#1

TS=(Thalamus) OR TS=(thalamic nuclei) OR ALL=(anterior thalamic nuclei) OR ALL=( geniculate bodies) OR ALL=(intralaminar thalamic nuclei) OR ALL=( lateral thalamic nuclei) OR ALL=( mediodorsal thalamic nucleus) OR ALL=(posterior thalamic nuclei) OR ALL=( ventral thalamic nuclei) OR ALL =(centromedian nucleus thalamus) OR ALL=(midline thalamic nuclei)

#2

TS=(Arousal) OR TS=(awareness) OR TS=(consciousness) OR ALL=(wakefulness)

#1 AND #2 AND LA=(English)

***CINAHL COMPLETE*** ***(advanced search)***

#1

TX thalamus OR TX thalamic nuclei OR TX anterior thalamic nuclei OR TX geniculate bodies OR TX intralaminar thalamic nuclei OR TX lateral thalamic nuclei OR TX mediodorsal thalamic nuclei OR TX posterior thalamic nuclei OR TX ventral thalamic nuclei OR TX centromedian nucleus thalamus OR TX midline thalamic nuclei

# 2

TX arousal OR TX awareness OR TX consciousness OR TX wakefulness

#1 AND # 2 AND AND LA English

# Supplementary Figure S1

**Supplementary Figure S1.** The figure shows an interactive plot representing the number of retrieved evidence for each thalamic nuclear group and their specific nuclei.

*Note: CM-Pf: centromedian-Parafascicular-complex; CL: central lateral nucleus; CeM: central medial nucleus; Pc: paracentral nucleus; VA: ventral anterior nucleus; VAmc: ventral anterior magnocellular division; VL: ventral lateral nucleus; VLa: ventral lateral anterior part; VLp: ventral lateral posterior part; VP: ventral posterior complex; VPM: ventral posteromedial nucleus; VPL: ventral posterolateral nucleus; MD: Mediodorsal nucleus; MDmc: mediodorsal magnocellular part; MDpc: mediodorsal parvocellullar parts; MDpl: mediodorsal paralaminar part; PUL: pulvinar nucleus; PULa: pulvinar anterior part; PULl: pulvinar lateral part; PULm: pulvinar medial part; PULi: pulvinar inferior part; LD: laterodorsal; LP: lateral posterior; MG: medial geniculate body; LG: lateral geniculate body; Sg-Li: suprageniculates-limitans nucleus; Po: posterior nucleus; TRN: reticular nucleus; Ad: anterodorsal nucleus; Av: anteroventral nucleus; Pv: paraventricular; Pt: paratenial; Re: reuniens*

# Supplementary Figure S2


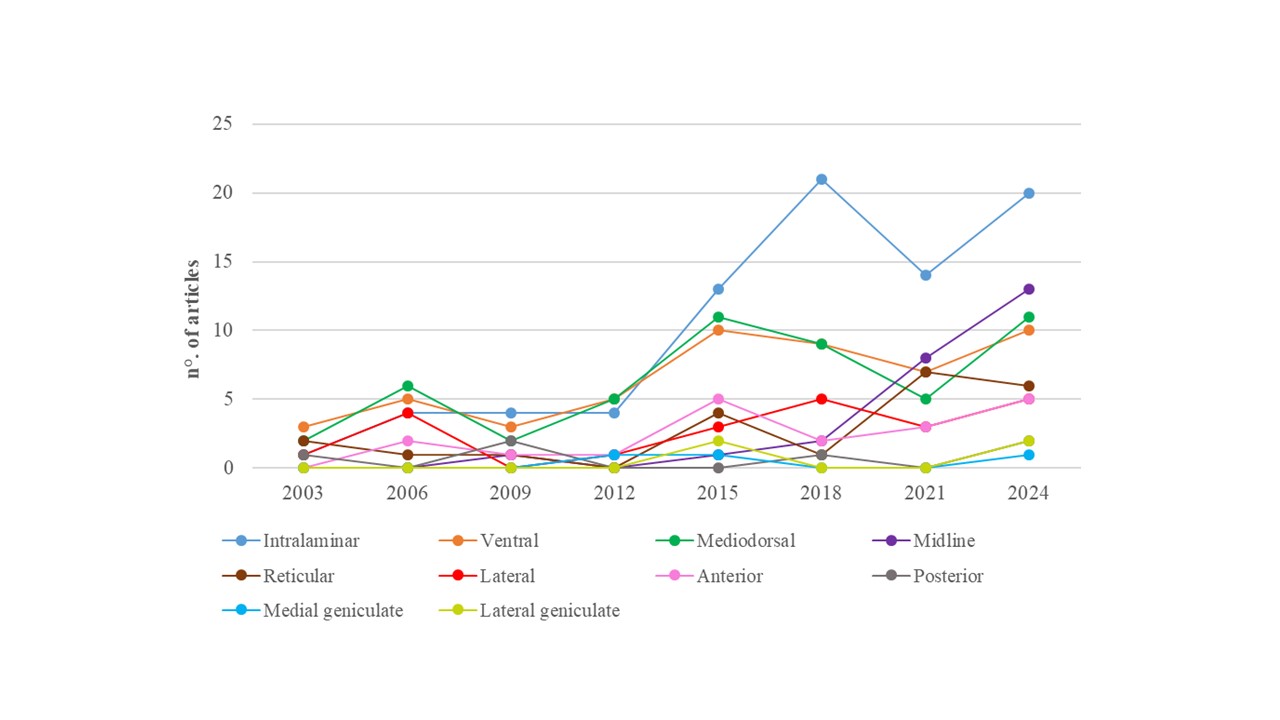


**Supplementary Figure S2.** The figure shows the number of retrieved studies (y-axis) for each thalamic nuclear group over the years (x-axis) covered in the present systematic review.

# Supplementary Table S1a.

For each thalamic nuclear group, the table shows the total number of pieces of evidence (2^nd^ column), number of positive pieces of evidence (3^rd^ column), and the numerical index used to identify the thalamic nuclear group most associated with the function of interest (4^th^ column; refer to the main text for the index computation) by considering the studies on animals.

| **Nuclear group** | **Tot_group_** | **Pos_group_** | **Index** |
| --- | --- | --- | --- |
| *Intralaminar* | 41 | 35 | 3.17 |
| *Midline* | 25 | 20 | 2.57 |
| *Reticular* | 20 | 17 | 2.54 |
| *Ventral* | 31 | 22 | 2.43 |
| *Mediodorsal* | 15 | 10 | 1.80 |
| *Posterior* | 5 | 4 | 1.28 |
| *Anterior* | 4 | 3 | 1.03 |
| *Lateral* | 2 | 2 | 0.69 |
| *Medial geniculate* | 1 | 1 | 0.00 |
| *Lateral geniculate* | 0 | 0 | - |

# Supplementary Table S1b.

For each thalamic nuclear group, the table shows the total number of pieces of evidence (2^nd^ column), number of positive pieces of evidence (3^rd^ column), and the numerical index used to identify the thalamic nuclear group most associated with the function of interest (4^th^ column; refer to the main text for the index computation) by considering the studies on human.

| **Nuclear group** | **Tot_group_** | **Pos_group_** | **Index** |
| --- | --- | --- | --- |
| *Intralaminar* | 61 | 58 | 3.90 |
| *Mediodorsal* | 38 | 35 | 3.35 |
| *Ventral* | 46 | 36 | 2.99 |
| *Lateral* | 30 | 21 | 2.38 |
| *Anterior* | 16 | 13 | 2.25 |
| *Midline* | 2 | 2 | 0.69 |
| *Reticular* | 2 | 2 | 0.69 |
| *Lateral geniculate* | 4 | 2 | 0.69 |
| *Medial geniculate* | 2 | 1 | 0.34 |
| *Posterior* | 1 | 1 | 0.00 |
|  |  |  |  |

# Supplementary Table S1c.

For each intralaminar nucleus, the table shows the total number of pieces of evidence (2^nd^ column), number of positive pieces of evidence (3^rd^ column), and the numerical index used to identify the intralaminar nucleus most associated with the function of interest (4^th^ column; refer to the main text for the index computation) by considering studies on animals.

| **Intralaminar nucleus** | **Tot_nucleus_** | **Pos_nucleus_** | **Index** |
| --- | --- | --- | --- |
| *Central lateral* | 15 | 13 | 2.34 |
| *Central medial* | 12 | 11 | 2.27 |
| *Centromedian-parafascicular complex* | 9 | 9 | 2.19 |
| *Paracentral* | 3 | 2 | 0.73 |

# Supplementary Table S1d.

For each intralaminar nucleus, the table shows the total number of pieces of evidence (2^nd^ column), number of positive pieces of evidence (3^rd^ column), and the numerical index used to identify the intralaminar nucleus most associated with the function of interest (4^th^ column; refer to the main text for the index computation) by considering studies on human.

| **Intralaminar nucleus** | **Tot_nucleus_** | **Pos_nucleus_** | **Index** |
| --- | --- | --- | --- |
| *Centromedian-parafascicular complex* | 31 | 29 | 3.21 |
| *Central lateral* | 10 | 10 | 2.30 |
| *Central medial* | 3 | 3 | 1.09 |
| *Paracentral* | 1 | 1 | 0 |

# Supplementary Table S2

***Supplementary Table S2***

**Supplementary Table S2.** The table lists the studies providing evidence of a relationship between consciousness/arousal/wakefulness and the intralaminar nuclear group.

Ad: anterodorsal nucleus; Av: anteroventral nucleus; CeM: central medial nucleus; CL: central lateral nucleus; CM-Pf: centromedian-parafascicular-complex; LD: laterodorsal; LP: lateral posterior; MD: mediodorsal nucleus; MDmc: mediodorsal magnocellular part; MDpc: mediodorsal parvocellullar parts; MDpl: mediodorsal paralaminar part; Pc: paracentral nucleus; Po: posterior nucleus; Pt: paratenial; PUL: pulvinar nucleus; PULa: pulvinar anterior part; PULi: pulvinar inferior part; PULl: pulvinar lateral part; PULm: pulvinar medial part; Pv: paraventricular; Re: reuniens; Sg-Li: suprageniculates-limitans nucleus; TRN: reticular nucleus; VA: ventral anterior nucleus; VL: ventral lateral nucleus; VLa: ventral lateral anterior part; VLp: ventral lateral posterior part; VP: ventral posterior complex; VPL: ventral posterolateral nucleus; VPM: ventral posteromedial nucleus.

| **Authors** | **Year** | **Function** | **Population** | **Sample size** | **Lateralization** | **Evidence** | | **Other nuclei** | **Notes** |
| --- | --- | --- | --- | --- | --- | --- | --- | --- | --- |
|  |  |  |  |  |  | **Positive** | **Negative** |  |  |
| Jang et al. (61) | 2015 | Consciousness | Human | 24 | B | X |  | - |  |
| Jang et al. (62) | 2016 | Consciousness | Human | 37 | B | X |  | - |  |
| Jang et al. (63) | 2015 | Consciousness | Human | 1 | B | X |  | - |  |
| Zheng et al. (64) | 2017 | Consciousness | Human | 25 | B | X |  | VA; VL; MD |  |
| Jang et al. (65) | 2023 | Consciousness | Human | 41 | Mixed | X |  | - |  |
| Jang et al. (66) | 2024 | Consciousness | Human | 31 | B | X |  | - |  |
| Jang et al. (67) | 2023 | Consciousness | Human | 17 | - | X |  | - |  |
| Jang et al. (68) | 2016 | Consciousness | Human | 1 | B | X |  | - |  |
| Jang et al. (69) | 2019 | Consciousness | Human | 1 | B | X |  | - |  |
| Jang et al. (70) | 2020 | Consciousness | Human | 1 | B | X |  | - |  |
| Jang et al. (71) | 2016 | Consciousness | Human | 1 | B | X |  | - |  |
| Jang et al. (72) | 2017 | Conscioisness | Human | 1 | B | X |  | - |  |
| Akeju et al. (73) | 2014 | Consciousness | Human | 27 | B | X |  | VA; MDmc; PUL; VL |  |
| Lemaire et al. (74) | 2018 | Consciousness | Human | 5 | - | X |  | - |  |
| Magrassi et al. (75) | 2016 | Consciounsess | Human | 3 | B | X |  | - |  |
| Yatziv et al. (76) | 2020 | Wakefulness | Animal | 10 | R |  | X | Pt |  |
| Gao et al. (77) | 2019 | Arousal | Animal | 38 | - |  | X | Pt; Re |  |
| Crone et al. (78) | 2018 | Consciounsess | Human | 20 | B |  | X | - |  |
| *Centromedian-Parafascicular complex (CM-Pf)* | | | | | | | | | |
| Chudy et al. (49) | 2023 | Consciousness | Human | 32 | Mixed | X |  | - |  |
| Chudy et al. (53) | 2018 | Consciousness | Human | 14 | Mixed | X |  | - |  |
| Yamamoto et al. (47) | 2013 | Consciousness | Human | 122 | B | X |  | - |  |
| Schiff et al. (48) | 2007 | Consciousness | Human | 1 | B | X |  | CL; MDpl |  |
| He et al. (50) | 2023 | Consciousness | Human | 23 | B | X |  | - |  |
| Shu et al. (54) | 2023 | Consciousness | Human | 10 | Mixed | X |  | - |  |
| Yamamoto et al. (56) | 2005 | Consciousness | Human | 26 | B | X |  | - |  |
| Quinkert et al. (79) | 2012 | Arousal | Animal | 26 | B | X |  | CL; Pc; CeM; MD |  |
| Arnts et al. (55) | 2022 | Consciousness | Human | 1 | B | X |  | - |  |
| Raguž et al. (46) | 2021 | Consciousness | Human | 5 | Mixed | X |  |  |  |
| Zhang et al. (51) | 2024 | Consciousness | Animal | 10 | R | X |  | - |  |
| Baker et al. (57) | 2016 | Arousal | Animal | 2 | Mixed | X |  | CL; MD |  |
| Luppi et al. (80) | 2024 | Consciousness | Animal | 5 | R | X |  | VL |  |
| Bastos et al. (81) | 2021 | Consciousness | Animal | 4 | B | X |  | CL; VPL; MD |  |
| Tasserie et al. (82) | 2022 | Consciousness | Animal | 5 | R | X |  | VL |  |
| Redinbaugh et al. (83) | 2020 | Arousal | Animal | 2 | R | X |  | CL; MD |  |
| Welch et al. (84) | 2021 | Consciousness | Human | 1 | B | X |  | - |  |
| Velasco et al. (85) | 2006 | Consciousness | Human | 13 | Mixed | X |  | - |  |
| Kokkinos et al. (86) | 2020 | Consciousness | Human | 1 | B | X |  | VPL; VLp |  |
| Kundu et al. (87) | 2023 | Arousal | Human | 1 | L | X |  | - |  |
| Hect et al. (88) | 2022 | Arousal | Human | 1 | B | X |  | - |  |
| Kumral et al. (89) | 2015 | Consciousness | Human | 252 | B | X |  | CL; MD |  |
| Långsjö et al. (90) | 2016 | Consciousness | Human | 1 | B | X |  | CL; MD |  |
| Carrera et al. (91) | 2004 | Consciousness | Human | 21 | Mixed | X |  | CL; MD |  |
| Magrassi et al. (45) | 2018 | Consciousness | Human | 3 | B | X |  | CL; MDpl |  |
| Maxwell et al. (92) | 2006 | Consciousness | Human | 10 | - | X |  | MDmc; MDpc; CL; Pc; CeM; VP; LP |  |
| Fridman et al. (59) | 2014 | Consciousness | Human | 24 | B | X |  | - |  |
| Edlow et al. (52) | 2013 | Consciousness | Human | 1 | B | X |  | LP; PUL; TRN; CL; MD |  |
| Cui et al. (93) | 2018 | Consciousness | Human | 33 | B | X |  | VPL; PUL |  |
| Zhou et al. (94) | 2011 | Consciousness | Human | 7 | B | X |  | - |  |
| Laouchedi et al. (95) | 2015 | Consciousness | Human | 24 | B | X |  | PUL; TRN; VA; VL; VP; MD |  |
| Jiang et al. (96) | 2021 | Consciousness | Animal | - | - | X |  | - |  |
| Sukhotinsky et al. (97) | 2007 | Consciousness | Animal | - | B | X |  | CL; Pc; CeM; VL; TRN |  |
| Nir et al. (98) | 2022 | Consciousness | Human | 72 | B | X |  | VLa; MD |  |
| Liu et al. (99) | 2013 | Consciousness | Human | 8 | B | X |  | VL; VP; MD |  |
| Weiner et al. (33) | 2023 | Consciousness | Human | 11 | B | X |  | CL; CeM; PULa; PULl; PULm; PULi VPL; SG-Li; LP; LD; Av; VLp; VLa; VAmc; Va; MD; Re |  |
| Kempf et al. (58) | 2009 | Arousal | Human | 13 | Mixed | X |  | Ventral internediate |  |
| Setzer et al. (100) | 2022 | Arousal | Human | 27 | - | X |  | Av; VA; VLa; VLp; VPL; PUL; MD |  |
| Hindman et al. (101) | 2018 | Consciousness | Human | 33 | B |  | X | - |  |
| Valentin et al. (102) | 2012 | Consciousness | Human | 1 | B |  | X | - |  |
| *Central Lateral (CL)* | | | | | | | | | |
| Quinkert et al. (79) | 2012 | Arousal | Animal | 26 | B | X |  | CM-Pf; Pc; CeM; MD |  |
| Baker et al. (57) | 2016 | Arousal | Animal | 2 | Mixed | X |  | CM-Pf; MD |  |
| Bastos et al. (81) | 2021 | Consciousness | Animal | 4 | B | X |  | CM-Pf VPL; MD |  |
| Gummadavelli et al. (103) | 2015 | Consciousness | Animal | - | B | X |  | - |  |
| Redinbaugh et al. (83) | 2020 | Arousal | Animal | 2 | R | X |  | CM-Pf; MD |  |
| Afrasiabi et al. (104) | 2021 | Consciousness | Animal | 2 | R | X |  | - |  |
| Xu et al. (105) | 2020 | Arousal | Animal | 21 | B | X |  | - |  |
| Claar et al. (106) | 2023 | Consciousness | Animal | - | - | X |  | Re; Av; MD; Po; VA; VPL; VPM |  |
| Redinbaugh et al. (107) | 2022 | Arousal | Animal | 2 | R | X |  | - |  |
| Liu et al. (108) | 2015 | Consciousness | Animal | 16 | R | X |  | Pc |  |
| Schiff et al. (48) | 2007 | Consciousness | Human | 1 | B | X |  | CM-Pf; MDpl |  |
| Gottshall et al. (109) | 2019 | Consciousness | Human | 1 | B | X |  | - |  |
| Kumral et al. (89) | 2015 | Consciousness | Human | 252 | B | X |  | CL; MD |  |
| Långsjö et al. (90) | 2016 | Consciousness | Human | 1 | B | X |  | CM-Pf; MD |  |
| Carrera et al. (91) | 2004 | Consciousness | Human | 21 | Mixed | X |  | CM-Pf; MD |  |
| Magrassi et al. (45) | 2018 | Consciousness | Human | 3 | B | X |  | CM-Pf; MDpl |  |
| Maxwell et al. (92) | 2006 | Consciousness | Human | 10 | - | X |  | MDmc; MDpc; CM-Pf; Pc; CeM; VP; LP |  |
| Edlow et al. (52) | 2013 | Consciousness | Human | 1 | B | X |  | CM-Pf; LP; PUL; TRN; MD |  |
| Motelow et al. (110) | 2015 | Consciousness | Human | 10 | B | X |  | - |  |
| Feng et al. (111) | 2017 | Consciousness | Animal | 38 | - | X |  | VPM |  |
| Flores et al. (112) | 2017 | Consciousness | Animal | - | - | X |  | LD; MD |  |
| Weiner et al. (33) | 2023 | Consciousness | Human | 11 | B | X |  | CM-Pf; CeM; PULa; PULl; PULm; PULim VPL; SG-Li; LP; LD; Av; VLp; VLa; VAmc; Va; MD; Re |  |
| Sukhotinsky et al. (97) | 2007 | Consciousness | Animal | - | B | X |  | CM-Pf; Pc; CeM; VL; TRN |  |
| Kundishora et al. (113) | 2017 | Arousal | Animal | - | B |  | X | - |  |
| González et al. (114) | 2019 | Consciousness | Animal | 25 | B |  | X | - |  |
| *Central Medial (CeM)* | | | | | | | | | |
| Maxwell et al. (92) | 2006 | Consciousness | Human | 10 | - | X |  | CM-Pf; Pc; CL; MDmc; MDpc; VP; LP |  |
| Weiner et al. (33) | 2023 | Consciousness | Human | 11 | B | X |  | CM-Pf; CL; PULa; PULl; PULm; PULim VPL; SG-Li; LP; LD; Av; VLp; VLa; VAmc; VA; MD; Re |  |
| Kantonen et al. (115) | 2023 | Consciousness | Human | 160 | L | X |  | - |  |
| Muheyati et al. (116) | 2024 | Consciousness | Animal | 285 | - | X |  | Pv; MD |  |
| Fu et al. (117) | 2017 | Consciousness | Animal | 131 | - | X |  | - |  |
| Baker et al. (118) | 2014 | Consciousness | Animal | 13 | - | X |  | - |  |
| Dringenberg et al. (119) | 2003 | Wakefulness | Animal | - | Mixed | X |  | CeM; TRN; MD |  |
| Alkire et al. (120) | 2009 | Consciousness | Animal | 106 | - | X |  | VL |  |
| Lioudyno et al. (121) | 2013 | Consciousness | Animal | - | - | X |  | - |  |
| Ramadasan-Nair et al. (122) | 2017 | Consciousness | Animal | - | B | X |  | MD |  |
| Quinkert et al. (79) | 2012 | Arousal | Animal | 26 | B | X |  | CM-Pf; Pc; CL; MD |  |
| Abe et al. (123) | 2017 | Consciousness | Animal | 131 | - | X |  | - |  |
| Gent et al. (124) | 2018 | Wakefulness | Animal | - | B | X |  | Re; Pv; VP; Ad; MD |  |
| Di Ianni et al. (125) | 2023 | Arousal | Animal | - | - | X |  | - |  |
| Sukhotinsky et al. (97) | 2007 | Consciousness | Animal | - | B |  | X | CL; Pc; CM-Pf; VL; TRN |  |
| *Paracentral (Pc)* | | | | | | | | | |
| Maxwell et al. (92) | 2006 | Consciousness | Human | 10 | - | X |  | CM-Pf; CeM; CL; MDmc; MDpc; VP; LP |  |
| Sukhotinsky et al. (97) | 2007 | Consciousness | Animal | - | B |  | X | CL; CeM; CM-Pf; VL; TRN |  |
| Quinkert et al. (79) | 2012 | Arousal | Animal | 26 | B | X |  | CM-Pf; CeM; CL; MD |  |
| Liu et al. (108) | 2015 | Consciousness | Animal | 16 | R | X |  | CL |  |

# Supplementary Table S3

***Supplementary Table S3***

**Supplementary Table S3.** The table lists the studies providing evidence of a relationship between consciousness/arousal/wakefulness and the mediodorsal nuclear group.

Ad: anterodorsal nucleus; Av: anteroventral nucleus; CeM: central medial nucleus; CL: central lateral nucleus; CM-Pf: centromedian-parafascicular-complex; LD: laterodorsal; LP: lateral posterior; MD: mediodorsal nucleus; MDmc: mediodorsal magnocellular part; MDpc: mediodorsal parvocellullar parts; MDpl: mediodorsal paralaminar part; Pc: paracentral nucleus; Po: posterior nucleus; Pt: paratenial; PUL: pulvinar nucleus; PULa: pulvinar anterior part; PULi: pulvinar inferior part; PULl: pulvinar lateral part; PULm: pulvinar medial part; Pv: paraventricular; Re: reuniens; Sg-Li: suprageniculates-limitans nucleus; TRN: reticular nucleus; VA: ventral anterior nucleus; VL: ventral lateral nucleus; VLa: ventral lateral anterior part; VLp: ventral lateral posterior part; VP: ventral posterior complex; VPL: ventral posterolateral nucleus; VPM: ventral posteromedial nucleus.

| **Authors** | **Year** | **Function** | **Population** | **Sample size** | **Lateralization** | **Evidence** | | **Other nuclei** | **Notes** |  |
| --- | --- | --- | --- | --- | --- | --- | --- | --- | --- | --- |
|  |  |  |  |  |  | **Positive** | **Negative** |  |  |  |
| Lutkenhoff et al. (34) | 2013 | Consciousness | Human | 25 | B | X |  | LD; VA; VPL; VL; VPM; LP; PUL; Anterior nuclear group; Lateral geniculate body: Medial geniculate body |  |  |
| Fernández-Espejo et al. (126) | 2010 | Consciousness | Human | 9 | B | X |  | - |  |  |
| Edlow et al. (52) | 2013 | Consciousness | Human | 1 | B | X |  | CL; CM-Pf; TRN; LP; PUL; Anterior nuclear group; Lateral geniculate body |  |  |
| Maxwell et al. (92) | 2006 | Consciousness | Human | 10 | - | X |  | CeM; CL; Pc; CM-Pf; LP; VP | Involvement of MDmc and MDpc |  |
| Graham et al. (127) | 2005 | Consciousness | Human | 35 | - | X |  | VP; LP |  |  |
| Juengling et al. (128) | 2005 | Consciousness | Human | 5 | B | X |  | - |  |  |
| Maxwell et al. (129) | 2004 | Consciousness | Human | 10 | L | X |  | VP; LP |  |  |
| Yu et al. (130) | 2021 | Consciousness | Human | 10 | Mixed | X |  | VA; Anterior nuclear group |  |  |
|  |  |  |  |  |  |  |  |  |  |  |
| Magrassi et al. (45) | 2018 | Consciousness | Human | 3 | B | X |  | CL; CM-Pf | Involvement of MDpl |  |
| Kumral et al. (89) | 2015 | Consciousness | Human | 252 | B | X |  | CM-Pf; CL; Anterior nuclear group |  |  |
| Långsjö et al. (90) | 2016 | Consciousness | Human | 1 | B | X |  | CM-Pf; CL |  |  |
| Carrera et al. (91) | 2004 | Consciousness | Human | 21 | Mixed | X |  | CM-Pf; CL |  |  |
| Perren et al. (131) | 2005 | Consciousness | Human | 12 | Mixed | X |  | LD; VA; VL; Anterior nuclear group |  |  |
| Zheng et al. (64) | 2017 | Consciousness | Human | 25 | B | X |  | VA; VL; PUL; Intralaminar nuclear group |  |  |
| Monti et al. (132) | 2015 | Consciousness | Human | 28 | - | X |  | VA |  |  |
| Cosgrove et al. (133) | 2022 | Consciousness | Human | 25 | B | X |  | - |  |  |
| Pozeg et al. (134) | 2023 | Consciousness | Human | 40 | L | X |  | PULm; VPL; VLp; VL; PUL |  |  |
| He et al. (135) | 2015 | Consciousness | Human | 9 | B | X |  | - |  |  |
| Yeh et al. (136) | 2013 | Consciousness | Human | 1 | B | X |  | - |  |  |
| Xu et al. (137) | 2023 | Consciousness | Human | 1 | B | X |  | - |  |  |
| Flores et al. (112) | 2017 | Consciousness | Animal | - | - | X |  | CL; LD; Posterior nuclear group |  |  |
| Choi et al. (138) | 2015 | Consciousness | Animal | 44 | - | X |  | - |  |  |
| Ramadasan-Nair et al. (122) | 2017 | Consciousness | Animal | - | B | X |  | CeM |  |  |
| Långsjö et al. (139) | 2012 | Consciousness | Human | 20 | B | X |  | - |  |  |
| Xie et al. (140) | 2011 | Consciousness | Human | 7 | B | X |  | - |  |  |
| Han et al. (141) | 2022 | Arousal | Human | 213 | B | X |  | VPL, PUL, MD |  |  |
| Weiner et al. (33) | 2023 | Consciousness | Human | 11 | B | X |  | PULl; PULa; PULi; PULm; VPL; Sg-Li; LP; LD; Av; CM-Pf; VLp; VLa; Re; CeM; VAmc; VA; CL; Medial geniculate body; Lateral geniculate body |  |  |
| Akeju et al. (73) | 2014 | Consciousness | Human | 27 | B | X |  | VA; VL; PUL; Intralaminar nuclear group; Midline nuclear group | Involvement of MDmc |  |
| Liu et al. (99) | 2013 | Consciousness | Human | 8 | B |  | X | CM-Pf; VL; VP; LD; CL |  |  |
| Smith et al. (142) | 2017 | Consciousness | Animal | 16 | R | X |  | - |  |  |
| Stamatakis et al. (143) | 2010 | Consciousness | Human | 16 | B | X |  | Anterior nuclear group |  |  |
| Iidaka et al. (144) | 2021 | Araousal | Human | 20 | B | X |  | PUL; VL | Involvement of MDmc and MDpc |  |
| Dringenberg t al. (119) | 2003 | Wakefulness | Animal | - | Mixed | X |  | CeM; TRN |  |  |
| Baker et al. (57) | 2016 | Araousal | Animal | 2 | Mixed | X |  | CL; CM-Pf |  |  |
| Bastos et al. (81) | 2021 | Consciousness | Animal | 4 | B | X |  | CL; CM-Pf; VPL |  |  |
| Quinkert et al. (79) | 2012 | Araousal | Animal | 26 | B | X |  | CL; Pc; CeM; CM-Pf |  |  |
| Schiff et al. (48) | 2007 | Consciousness | Human | 1 | B | X |  | CL; CM-Pf | Involvement of MDpl |  |
| Claar et al. (106) | 2023 | Consciousness | Animal | - | - | X |  | CL; TRN; Av; Po; VA; VPL; VPM |  |  |
| Sriji et al. (145) | 2021 | Wakefulness | Animal | 19 | B | X |  | - |  |  |
| Gent et al. (124) | 2018 | Wakefulness | Animal | - | B |  | X | CeM; Ad; Re; Pv; VP; |  |  |
| Angelakos et al. (146) | 2023 | Wakefulness | Animal | - | - |  | X | Pv |  |  |
| Leeman-Markowski et al. (147) | 2015 | Consciousness | Human | 1 | L | X |  | - |  |  |
| Rennebaum et al. (148) | 2016 | Consciousness | Human | 69 | - | X |  | PUL |  |  |
| Salek-Haddadi et al. (149) | 2003 | Consciousness | Human | 1 | B | X |  | VPL; VL |  |  |
| Kundu et al. (87) | 2023 | Consciousness | Human | 1 | L | X |  | A; CM-Pf; PUL |  |  |
| Setzer et al. (100) | 2022 | Araousal | Human | 27 | - | X |  | CM-Pf; VPL; Av; PUL; VA; VPL; VLa; VLp; Lateral geniculate body |  |  |
| Englot et al. (150) | 2009 | Consciousness | Animal | 109 | R |  | X | - |  |  |
| Redinbaugh et al. (83) | 2020 | Araousal | Animal | 2 | R |  | X | CL; CM-Pf |  |  |
| Muheyati et al. (116) | 2024 | Consciousness | Animal | 285 | - |  | X | CeM; Pv |  |  |
| Nir et al. (98) | 2022 | Consciousness | Human | 72 | B |  | X | CM-Pf; VLa |  |  |
| Laouchedi et al. (95) | 2015 | Consciousness | Human | 24 | B |  | X | CM-Pf; TRN; VA; VL; VP; PUL; Anterior nuclear group |  |  |

# Supplementary Table S4

***Supplementary Table S4***

**Supplementary Table S4.** The table lists the studies providing evidence of a relationship between consciousness/arousal/wakefulness and the ventral nuclear group.

Ad: anterodorsal nucleus; Av: anteroventral nucleus; CeM: central medial nucleus; CL: central lateral nucleus; CM-Pf: centromedian-parafascicular-complex; LD: laterodorsal; LP: lateral posterior; MD: mediodorsal nucleus; MDmc: mediodorsal magnocellular part; MDpc: mediodorsal parvocellullar parts; MDpl: mediodorsal paralaminar part; Pc: paracentral nucleus; Po: posterior nucleus; Pt: paratenial; PUL: pulvinar nucleus; PULa: pulvinar anterior part; PULi: pulvinar inferior part; PULl: pulvinar lateral part; PULm: pulvinar medial part; Pv: paraventricular; Re: reuniens; Sg-Li: suprageniculates-limitans nucleus; TRN: reticular nucleus; VA: ventral anterior nucleus; VL: ventral lateral nucleus; VLa: ventral lateral anterior part; VLp: ventral lateral posterior part; VP: ventral posterior complex; VPL: ventral posterolateral nucleus; VPM: ventral posteromedial nucleus.

| **Authors** | **Year** | **Function** | **Population** | **Sample size** | **Lateralization** | **Evidence** | | **Other nuclei** | **Notes** |
| --- | --- | --- | --- | --- | --- | --- | --- | --- | --- |
|  |  |  |  |  |  | **Positive** | **Negative** |  |  |
| Kempf et al. (58) | 2009 | Arousal | Human | 13 | Mixed | X |  | CM-Pf |  |
| Bridoux et al. (151) | 2015 | Wakefulness | Human | 11 | B | X |  | - |  |
| Malekmohammadi et al. (152) | 2019 | Consciousness | Human | 10 | Mixed | X |  | - |  |
| *Ventral Posterior Complex (VP)* | | | | | | | | | |
| Graham et al. (127) | 2005 | Consciousness | Human | 35 | B | X |  | LP; MD |  |
| Maxwell et al. (129) | 2004 | Consciousness | Human | 10 | L | X |  | LP; MD |  |
| Laouchedi et al. (95) | 2015 | Consciousness | Human | 24 | B | X |  | VA; VL; MD; CM-Pf; TRN; PUL |  |
| Maxwell et al. (92) | 2006 | Consciousness | Human | 10 | - |  | X | MDpc; MDmc; CL; Pc; CeM; LP; CM-Pf |  |
| Liu et al. (99) | 2013 | Consciousness | Human | 8 | B |  | X | CM-Pf; VL; MD |  |
| Kim et al. (153) | 2012 | Consciousness | Animal | 10 | - |  | X | VL |  |
| Gent et al. (124) | 2018 | Wakefulness | Animal | - | B |  | X | CeM; Re; Pv; Ad; MD |  |
| Ying et al. (22) | 2005 | Consciousness | Animal | - | - | X |  | - |  |
| Andrada et al. (154) | 2012 | Consciousness | Animal | 5 | - | X |  | Medial geniculate body | Involvement of VPL and VPM |
| Claar et al. (106) | 2023 | Consciousness | Animal | - | - | X |  | CL; TRN; Av; MD; Po; VA | Involvement of VPM and VPL |
| Cui et al. (93) | 2018 | Consciousness | Human | 33 | B | X |  | PUL; CM-Pf | Involvement of VPL |
| Pozeg et al. (134) | 2023 | Consciousness | Human | 40 | L | X |  | PUL; PULm; VLp; MD | Involvement of VPL |
| Weiner et al. (33) | 2023 | Consciousness | Human | 11 | B | X |  | PULl; PULm; PULa; PULi; VLa; VLp; Sg-Li; LP; LD; CM-Pf; CL; Av; VAmc; Re; CeM; MD | Involvement of VPL |
| Verdonck et al. (155) | 2014 | Consciousness | Human | 3 | L | X |  | - | Involvement of VPL |
| Chen et al. (156) | 2012 | Consciousness | Animal | 1 | - | X |  | - | Involvement of VPL |
| Bastos et al. (81) | 2021 | Consciousness | Animal | 4 | B | X |  | CL; CM-Pf; MD | Involvement of VPL |
| Kokkinos et al. (86) | 2020 | Consciousness | Human | 1 | B | X |  | CM-Pf; VLp | Involvement of VPL |
| Salek-Haddadi et al. (149) | 2003 | Consciousness | Human | 1 | B | X |  | MD; VL | Involvement of VPL |
| Setzer et al. (100) | 2022 | Arousal | Human | 16 | - | X |  | Av; CM-Pf; PUL; VA; VLa; VLp; MD | Involvement of VPL |
| Han et al. (141) | 2024 | Arousal | Human | 213 | B | X |  | PUL; MD | Involvement of VPL |
| Reed et al. (157) | 2015 | Consciousness | Animal | 9 | - | X |  | - | Involvement of VPM |
| Hu et al. (158) | 2023 | Consciousness | Animal | - | - | X |  | Pv | Involvement of VPM |
| Plourde et al. (159) | 2017 | Consciousness | Animal | 10 | - | X |  | - | Involvement of VPM |
| Yang et al. (160) | 2019 | Consciousness | Animal | 48 | B | X |  | TRN | Involvement of VPM |
| Hwang et al. (161) | 2012 | Consciousness | Animal | 10 | - | X |  | VL | Involvement of VPM |
| Plourde et al. (163) | 2013 | Consciousness | Animal | 9 | - | X |  | - | Involvement of VPM |
| Reed et al. (162) | 2013 | Consciousness | Animal | 10 | - | X |  | - | Involvement of VPM |
| Feng et al. (111) | 2017 | Consciousness | Animal | 38 | - | X |  | CL | Involvement of VPM |
| Tenney et al. (164) | 2003 | Consciousness | Animal | 8 | B | X |  | TRN; LD | Involvement of VPL and VPM |
| Zobeiri et al. (165) | 2019 | Wakefulness | Animal | - | - | X |  | - | Involvement of VPM |
| Lutkenhoff et al. (34) | 2013 | Consciousness | Human | 25 | B |  | X | VA; VL; LD; LP; MD; PUL | Involvement of VPL and VPM |
| Jiang et al. (166) | 2023 | Wakefulness | Animal | 11 | - |  | X | TRN | Involvement of VPM |
| Honjoh et al. (167) | 2018 | Wakefulness | Animal | - | B |  | X | - | Involvement of VPM |
| Silva et al. (168) | 2010 | Consciousness | Animal | 5 | - |  | X | - | Involvement of VPL |
| Abe et al. (123) | 2017 | Consciousness | Animal | 131 | - |  | X | CeM | Involvement of VPL |
| *Ventral Lateral (VL)* | | | | | | | | | |
| Perren et al. (131) | 2005 | Consciousness | Human | 12 | Mixed | X |  | VA; LD; MD |  |
| Lutkenhoff et al. (34) | 2013 | Consciousness | Human | 25 | B | X |  | VA; VPL; VPM; LD; LP; MD; PUL |  |
| Pozeg et al. (134) | 2023 | Consciousness | Human | 40 | L | X |  | PUL; PULm; VPL; MD | Involvement of VLp |
| Zheng et al. (64) | 2017 | Consciousness | Human | 25 | B | X |  | VA; MD; PUL |  |
| Laouchedi et al. (95) | 2015 | Consciousness | Human | 24 | B | X |  | VA; VPL; MD; CM-Pf; TRN; PUL |  |
| Thibaut et al. (169) | 2015 | Consciousness | Human | 21 | L | X |  | - |  |
| White et al. (170) | 2003 | Consciousness | Human | 11 | L | X |  | VA |  |
| Hwang et al. (161) | 2012 | Consciousness | Animal | 10 | - | X |  | VPM |  |
| Kim et al. (153) | 2012 | Consciousness | Animal | 10 | - | X |  | VP |  |
| Akeju et al. (73) | 2014 | Consciousness | Human | 17 | B | X |  | VA; MDmc; PULm |  |
| Weiner et al. (33) | 2023 | Consciousness | Human | 11 | B | X |  | PULl; PULm; PULa; PULi; VPL; Sg-Li; LP; LD; CM-Pf; CL; Av; VAmc; Re; CeM; MD | Involvement of VLp and VLa |
| Sukhotinsky et al. (97) | 2007 | Consciousness | Animal | - | B | X |  | CM-Pf; CL; Pc; CeM; TRN |  |
| Salek-Haddadi et al. (149) | 2003 | Consciousness | Human | 1 | B | X |  | VPL; MD |  |
| Kokkinos et al. (86) | 2020 | Consciousness | Human | 1 | B | X |  | CM-Pf; VPL | Involvement of VLp |
| Luppi et al. (80) | 2024 | Consciousness | Animal | 5 | R |  | X | CM-Pf |  |
| Tasserie et al. (82) | 2022 | Consciousness | Animal | 5 | R |  | X | CM-Pf |  |
| Liu et al. (99) | 2013 | Consciousness | Human | 8 | B |  | X | CM-Pf; VLP; MD |  |
| Nir et al. (98) | 2022 | Consciousness | Human | 72 | B |  | X | CM-Pf; MD | Involvement of VLa |
| Alkire et al. (120) | 2009 | Consciousness | Animal | 106 | - |  | X | CM-Pf |  |
| Setzer et al. (100) | 2022 | Arousal | Human | 13 | - |  | X | Av; CM-Pf; PUL; VA; VPL; MD | Involvement of VLp and VLa |
| Iidaka (144) | 2021 | Arousal | Human | 20 | B |  | X | PUL; MDmc; MDpc |  |
| *Ventral Anterior (VA)* | | | | | | | | | |
| Perren et al. (131) | 2005 | Consciousness | Human | 12 | Mixed | X |  | VL; LD; MD |  |
| Zheng et al. (64) | 2017 | Consciousness | Human | 25 | B | X |  | VL; MD; PUL |  |
| Monti et al. (132) | 2015 | Consciousness | Human | 28 | - | X |  | MD |  |
| Yu et al. (130) | 2021 | Consciousness | Human | 10 | Mixed | X |  | MD |  |
| Lutkenhoff et al. (34) | 2013 | Consciousness | Human | 25 | B | X |  | VL; VPL; VPM; LD; LP; MD; PUL |  |
| Laouchedi et al. (95) | 2015 | Consciousness | Human | 24 | B | X |  | VL; VPL; MD; CM-Pf; TRN; PUL |  |
| Cheng et al. (171) | 2018 | Consciousness | Human | 3 | B | X |  | - |  |
| Akeju et al. (73) | 2014 | Consciousness | Human | 17 | B | X |  | VL; MDmc; PULm |  |
| White et al. (170) | 2003 | Consciousness | Human | 11 | L | X |  | VL |  |
| Weiner et al. (33) | 2023 | Consciousness | Human | 11 | B | X |  | PULl; PULm; PULa; PULi; VPL; Sg-Li; LP; LD; CM-Pf; CL; Av; VLp; VLa; Re; CeM; MD | Involvement of VAmc |
| Claar et al. (106) | 2023 | Consciousness | Animal | - | - | X |  | CL; TRN; Av; MD; Po; VPL; VPM |  |
| Setzer et al. (100) | 2022 | Arousal | Human | 13 | - |  | X | Av; CM-Pf; PUL; VLa; VLp; VPL; MD |  |

# Supplementary Table S5

***Supplementary Table S5***

**Supplementary Table S5.** The table lists the studies providing evidence of a relationship between consciousness/arousal/wakefulness and the midline nuclear group.

Ad: anterodorsal nucleus; Av: anteroventral nucleus; CeM: central medial nucleus; CL: central lateral nucleus; CM-Pf: centromedian-parafascicular-complex; LD: laterodorsal; LP: lateral posterior; MD: mediodorsal nucleus; MDmc: mediodorsal magnocellular part; MDpc: mediodorsal parvocellullar parts; MDpl: mediodorsal paralaminar part; Pc: paracentral nucleus; Po: posterior nucleus; Pt: paratenial; PUL: pulvinar nucleus; PULa: pulvinar anterior part; PULi: pulvinar inferior part; PULl: pulvinar lateral part; PULm: pulvinar medial part; Pv: paraventricular; Re: reuniens; Sg-Li: suprageniculates-limitans nucleus; TRN: reticular nucleus; VA: ventral anterior nucleus; VL: ventral lateral nucleus; VLa: ventral lateral anterior part; VLp: ventral lateral posterior part; VP: ventral posterior complex; VPL: ventral posterolateral nucleus; VPM: ventral posteromedial nucleus.

| **Authors** | **Year** | **Function** | **Population** | **Sample size** | **Lateralization** | **Evidence** | | **Other nuclei** | **Notes** |
| --- | --- | --- | --- | --- | --- | --- | --- | --- | --- |
|  |  |  |  |  |  | **Positive** | **Negative** |  |  |
| Akeju et al. (73) | 2014 | Consciousness | Human | 27 | B | X |  | MDmc; VA; VL; PUL; Intralaminar nuclear group |  |
| Sukhotinsky et al. (97) | 2007 | Consciousness | Animal | - | B |  | X | CM-Pf; CL; Pc; VL; TRN; CeM; Posterior nuclear group; Anterior nuclear group |  |
| *Paraventricular (Pv)* | | | | | | | | | |
| Ren et al. (25) | 2018 | Wakefulness | Animal | - | B | X |  | - |  |
| Zhao et al. (172) | 2021 | Consciousness | Animal | - | - | X |  | - |  |
| Ao et al. (173) | 2021 | Consciousness | Animal | - | - | X |  | - |  |
| Li et al. (174) | 2022 | Consciousness | Animal | 225 | B | X |  | - |  |
| Wang et al. (175) | 2023 | Consciousness | Animal | - | - | X |  | - |  |
| Zhao et al. (176) | 2024 | Consciousness | Animal | - | - | X |  | - |  |
| Duan et al. (177) | 2024 | Consciousness | Animal | - | - | X |  | - |  |
| Wu et al. (178) | 2024 | Consciousness | Animal | - | - | X |  | - |  |
| Muheyati et al. (116) | 2024 | Consciousness | Animal | 285 | - | X |  | CeM; MD |  |
| Bu et al. (179) | 2022 | Consciousness | Animal | - | - | X |  | - |  |
| Zhao et al. (180) | 2023 | Consciousness | Animal | - | B | X |  | - |  |
| Liu et al. (181) | 2022 | Arousal | Animal | - | - | X |  | - |  |
| Ren et al. (182) | 2024 | Wakefulness | Animal | - | - | X |  | - |  |
| Gao et al. (183) | 2020 | Wakefulness | Animal | - | - | X |  | - |  |
| Liu et al. (184) | 2021 | Consciousness | Animal | 40 | - | X |  | - |  |
| Hu et al. (158) | 2023 | Consciousness | Animal | - | - | X |  | VPM |  |
| Gao et al. (77) | 2019 | Arousal | Animal | 38 | - | X |  | Re; Intralaminar nuclear group |  |
| Ao et al. (185) | 2021 | Consciousness | Animal | 34 | B | X |  | - |  |
| Yatziv et al. (76) | 2020 | Wakefulness | Animal | 10 | R | X |  | Intralaminar nuclear group |  |
| Gent et al. (124) | 2018 | Wakefulness | Animal | - | - |  | X | CeM; Ad; MD; VP; Re |  |
| Angelakos et al. (146) | 2023 | Wakefulness | Animal | - | - |  | X | MD |  |
| *Reuniens (Re)* | | | | | | | | | |
| Weiner et al. (33) | 2023 | Consciousness | Human | 11 | B | X |  | MD; PULl; PULa; PULi; PULm; VPL; Sg-Li; LP; LD; Av; CM-Pf; VLp; VLa; CeM; VAmc; VA; CL; Medial geniculate body; Lateral geniculate body |  |
| Gao et al. (77) | 2019 | Arousal | Animal | - | - |  | X | Pv; Intralaminar nuclear group |  |
| Gent et al. (124) | 2018 | Wakefulness | Animal | - | - |  | X | CeM; Ad; MD; VP; Pv |  |
| *Paratenial (Pt)* | | | | | | | | | |
| Zhao et al. (186) | 2020 | Consciousness | Animal | 6 | - | X |  | - |  |

# Supplementary Table S6

***Supplementary Table S6***

**Supplementary Table S6.** The table lists the studies providing evidence of a relationship between consciousness/arousal/wakefulness and the reticular nuclear group.

Ad: anterodorsal nucleus; Av: anteroventral nucleus; CeM: central medial nucleus; CL: central lateral nucleus; CM-Pf: centromedian-parafascicular-complex; LD: laterodorsal; LP: lateral posterior; MD: mediodorsal nucleus; MDmc: mediodorsal magnocellular part; MDpc: mediodorsal parvocellullar parts; MDpl: mediodorsal paralaminar part; Pc: paracentral nucleus; Po: posterior nucleus; Pt: paratenial; PUL: pulvinar nucleus; PULa: pulvinar anterior part; PULi: pulvinar inferior part; PULl: pulvinar lateral part; PULm: pulvinar medial part; Pv: paraventricular; Re: reuniens; Sg-Li: suprageniculates-limitans nucleus; TRN: reticular nucleus; VA: ventral anterior nucleus; VL: ventral lateral nucleus; VLa: ventral lateral anterior part; VLp: ventral lateral posterior part; VP: ventral posterior complex; VPL: ventral posterolateral nucleus; VPM: ventral posteromedial nucleus.

| **Authors** | **Year** | **Function** | **Population** | **Sample size** | **Lateralization** | **Evidence** | | **Other nuclei** | **Notes** |
| --- | --- | --- | --- | --- | --- | --- | --- | --- | --- |
|  |  |  |  |  |  | **Positive** | **Negative** |  |  |
| Ying et al. (22) | 2005 | Consciousness | Animal | - | - | X |  | VP |  |
| Zhang et al. (187) | 2020 | Consciousness | Animal | - | - | X |  | - |  |
| Yang et al. (160) | 2019 | Consciousness | Animal | 48 | B | X |  | VPM |  |
| Jiang et al. (166) | 2023 | Wakefulness | Animal | 11 | - | X |  | VPM |  |
| Frasch et al. (188) | 2007 | Consciousness | Animal | 6 | L | X |  | - |  |
| Liu et al. (189) | 2021 | Consciousness | Animal | - | B | X |  | - |  |
| Liu et al. (20) | 2021 | Wakefulness | Animal | - | B | X |  | - |  |
| Herrera et al. (190) | 2016 | Wakefulness | Animal | - | B | X |  | - |  |
| Cai et al. (191) | 2023 | Consciousness | Animal | - | - | X |  | - |  |
| Lewis et al. (192) | 2015 | Arousal | Animal | - | L | X |  | - |  |
| Yi et al. (193) | 2024 | Consciousness | Animal | - | Mixed | X |  | - |  |
| Claar et al. (106) | 2023 | Consciousness | Animal | - | - | X |  | CL; Av; MD; Po; VA; VPL; VPM |  |
| Edlow et al. (52) | 2013 | Consciousness | Human | 1 | B | X |  | CL; CM-Pf; MD; LP; PUL; Anterior nuclear group |  |
| Pais-Roldan et al. (194) | 2019 | Consciousness | Animal | 32 | B | X |  | - |  |
| Laouchedi et al. (95) | 2015 | Consciousness | Human | 24 | B | X |  | CM-Pf; VA; VL; VP; PUL; MD; Anterior nuclear group |  |
| Tenney et al. (164) | 2003 | Consciousness | Animal | 8 | B | X |  | VPM; VPL; LD; Posterior nuclear group |  |
| Lee et al. (21) | 2014 | Consciousness | Animal | - | - | X |  | - |  |
| Chang et al. (195) | 2022 | Consciousness | Animal | - | B | X |  | - |  |
| Liu et al. (196) | 2023 | Consciousness | Animal | - | - | X |  | - |  |
| Mesbah-Oskui et al. (197) | 2021 | Consciousness | Animal | 16 | L |  | X | - |  |
| Dringenberg et al. (119) | 2003 | Wakefulness | Animal | - | Mixed |  | X | MD; CeM |  |
| Sukhotinsky et al. (97) | 2007 | Consciousness | Animal | - | B |  | X | CM-Pf; CL; Pc; VL; P; CeM; Anterior nuclear group; Midline nuclear group |  |

# Supplementary Table S7

***Supplementary Table S7***

**Supplementary Table S7.** The table lists the studies providing evidence of a relationship between consciousness/arousal/wakefulness and the lateral nuclear group.

Ad: anterodorsal nucleus; Av: anteroventral nucleus; CeM: central medial nucleus; CL: central lateral nucleus; CM-Pf: centromedian-parafascicular-complex; LD: laterodorsal; LP: lateral posterior; MD: mediodorsal nucleus; MDmc: mediodorsal magnocellular part; MDpc: mediodorsal parvocellullar parts; MDpl: mediodorsal paralaminar part; Pc: paracentral nucleus; Po: posterior nucleus; Pt: paratenial; PUL: pulvinar nucleus; PULa: pulvinar anterior part; PULi: pulvinar inferior part; PULl: pulvinar lateral part; PULm: pulvinar medial part; Pv: paraventricular; Re: reuniens; Sg-Li: suprageniculates-limitans nucleus; TRN: reticular nucleus; VA: ventral anterior nucleus; VL: ventral lateral nucleus; VLa: ventral lateral anterior part; VLp: ventral lateral posterior part; VP: ventral posterior complex; VPL: ventral posterolateral nucleus; VPM: ventral posteromedial nucleus.

| **Authors** | **Year** | **Function** | **Population** | **Sample size** | **Lateralization** | **Evidence** | | **Other nuclei** | **Notes** |
| --- | --- | --- | --- | --- | --- | --- | --- | --- | --- |
|  |  |  |  |  |  | **Positive** | **Negative** |  |  |
| *Pulvinar (PUL)* | | | | | | | | | |
| Edlow et al. (52) | 2013 | Consciousness | Human | 1 | B | X |  | CL; CM-Pf; TRN; MD; LP; Anterior nuclear group; Lateral geniculate body |  |
| Zheng et al. (64) | 2017 | Consciousness | Human | 25 | B | X |  | VA; VL; MD; Intralaminar nuclear group |  |
| Cui et al. (93) | 2018 | Consciousness | Human | 33 | B | X |  | VPL; CM-Pf |  |
| Pozeg et al. (134) | 2023 | Consciousness | Human | 40 | L | X |  | MD; VPL; VLp; VL | Involvement of PUL and PULm |
| Kamakura et al. (198) | 2016 | Consciousness | Human | 1 | L | X |  |  |  |
| Kondratyeva et al. (199) | 2020 | Consciousness | Human | 34 | B | X | X | Anterior nuclear group | Involvement of PULm (positive); PULl (negative) |
| Rennebaum et al. (148) | 2016 | Consciousness | Human | 69 | - | X |  | MD |  |
| Filipescu et al. (200) | 2019 | Consciousness | Human | 8 | Mixed | X |  | - | Involvement PULm |
| Kundu et al. (87) | 2023 | Consciousness | Human | 1 | L | X |  | CM-Pf; MD; Anterior nuclear group |  |
| Weiner et al. (33) | 2023 | Consciousness | Human | 11 | B | X |  | MD; VPL; Sg-Li; LP; LD; Av; CM-Pf; VLp; VLa; Re; CeM; VAmc; VA; CL; Medial geniculate body; Lateral geniculate body | Involvement PULl; PULa; PULi; PULm |
| Han et al. (141) | 2024 | Arousal | Human | 213 | B | X |  | VPL; MD |  |
| Iidaka et al. (144) | 2021 | Arousal | Human | 20 | B |  | X | MD; VL |  |
| Setzer et al. (100) | 2022 | Arousal | Human | 27 | - |  | X | CM-Pf; VPL; Av; MD; VA; VLa; VLp; Lateral geniculate body |  |
| Lutkenhoff et al. (34) | 2013 | Consciousness | Human | 25 | B |  | X | LD; MD; VA; VPL; VL; VPM; LP; Anterior nuclear group; Lateral geniculate body; Medial geniculate body |  |
| Laouchedi et al. (95) | 2015 | Consciousness | Human | 24 | B |  | X | CM-Pf; TRN; VA; VL; VP; MD; Anterior nuclear group |  |
| *Lateral Posterior (LP)* | | | | | | | | | |
| Vanhaudenhuyse et al. (201) | 2010 | Consciousness | Human | 14 | L | X |  | - |  |
| Edlow et al. (52) | 2013 | Consciousness | Human | 1 | B | X |  | CL; CM-Pf; TRN; MD; PUL; Anterior nuclear group; Lateral geniculate body |  |
| Weiner et al. (33) | 2023 | Consciousness | Human | 11 | B | X |  | MD; PULl; PULa; PULi; PULm; VPL; Sg-Li; LD; Av; CM-Pf; VLp; VLa; Re; CeM; VAmc; VA; CL; Medial geniculate body; Lateral geniculate body |  |
| Lutkenhoff et al. (34) | 2013 | Consciousness | Human | 25 | B |  | X | LD; MD; VA; VPL; VL; VPM; PUL; Anterior nuclear group; Medial geniculate body; Lateral geniculate body |  |
| Maxwell et al. (92) | 2006 | Consciousness | Human | 10 | - |  | X | CeM; CL; Pc; CM-Pf; MDmc; MDpc; VP |  |
| Graham et al. (127) | 2005 | Consciousness | Human | 35 | - |  | X | VP; MD |  |
| Maxwell et al. (129) | 2004 | Consciousness | Human | 10 | L |  | X | VP; MD |  |
| *Laterodorsal (LD)* | | | | | | | | | |
| Lutkenhoff et al. (34) | 2013 | Consciousness | Human | 25 | B | X |  | MD; VA; VPL; VL; VPL; LP; PUL; Anterior; Medial geniculate body; Lateral geniculate body |  |
| Perren et al. (131) | 2005 | Consciousness | Human | 12 | Mixed | X |  | MD; VA; VL; Anterior nuclear group |  |
| Tenney et al. (164) | 2003 | Consciousness | Animal | 8 | B | X |  | VPM; VPL; TRN; Posterior nuclear group |  |
| Weiner et al. (33) | 2023 | Consciousness | Human | 11 | B | X |  | MD; PULl; PULa; PULi; PULm; VPL; Sg-Li; LP; Av; CM-Pf; VLp; VLa; Re; CeM; VAmc; VA; CL; Medial geniculate body; Lateral geniculate body |  |
| Flores et al. (112) | 2017 | Consciousness | Animal | - | - | X |  | CL; MD; Posterior nuclear group |  |

# Supplementary Table S8

***Supplementary Table S8***

**Supplementary Table S8.** The table lists the studies providing evidence of a relationship between consciousness/arousal/wakefulness and the anterior nuclear group.

Ad: anterodorsal nucleus; Av: anteroventral nucleus; CeM: central medial nucleus; CL: central lateral nucleus; CM-Pf: centromedian-parafascicular-complex; LD: laterodorsal; LP: lateral posterior; MD: mediodorsal nucleus; MDmc: mediodorsal magnocellular part; MDpc: mediodorsal parvocellullar parts; MDpl: mediodorsal paralaminar part; Pc: paracentral nucleus; Po: posterior nucleus; Pt: paratenial; PUL: pulvinar nucleus; PULa: pulvinar anterior part; PULi: pulvinar inferior part; PULl: pulvinar lateral part; PULm: pulvinar medial part; Pv: paraventricular; Re: reuniens; Sg-Li: suprageniculates-limitans nucleus; TRN: reticular nucleus; VA: ventral anterior nucleus; VL: ventral lateral nucleus; VLa: ventral lateral anterior part; VLp: ventral lateral posterior part; VP: ventral posterior complex; VPL: ventral posterolateral nucleus; VPM: ventral posteromedial nucleus.

| **Authors** | | **Year** | **Function** | | **Population** | | **Sample size** | **Lateralization** | | **Evidence** | | | **Other nuclei** | | | **Notes** |
| --- | --- | --- | --- | --- | --- | --- | --- | --- | --- | --- | --- | --- | --- | --- | --- | --- |
|  |  |  |  |  |  |  |  |  |  | **Positive** | **Negative** | |  |  |  |  |
| Stamatakis et al. (143) | | 2010 | Consciousness | | Human | | 16 | B | | X |  | | MD | | |  |
| Sukhotinsky et al. (97) | | 2007 | Consciousness | | Animal | | - | B | | X |  | | CM-Pf; CL; Pc; VL; TRN; CeM; Posterior nuclear group; Midline nuclear group | | |  |
| Bucurenciu et al. (202) | | 2020 | Wakefulness | | Human | | 4 | B | | X |  | | - | | |  |
| Voges et al. (203) | | 2015 | Arousal | | Human | | 9 | - | | X |  | | - | | |  |
| Lee et al. (204) | | 2017 | Consciousness | | Human | | 1 | B | | X |  | | - | | |  |
| Kerrigan et al. (205) | | 2004 | Consciousness | | Human | | 5 | B | | X |  | | - | | |  |
| Kundu et al. (87) | | 2023 | Consciousness | | Human | | 1 | L | | X |  | | CM-Pf; MD; PUL | | |  |
| Singh et al. (206) | | 2024 | Consciousness | | Human | | 4 | Mixed | | X |  | | - | | |  |
| Lutkenhoff et al. (34) | | 2013 | Consciousness | | Human | | 25 | B | | X |  | | LD; MD; VA; VPL; VL; VPM; LP; PUL; Lateral geniculate body; Medial geniculate body | | |  |
| Perren et al. (131) | | 2005 | Consciousness | | Human | | 12 | Mixed | | X |  | | MD; LD; VA; VL | | |  |
| Edlow et al. (52) | | 2013 | Consciousness | | Human | | 1 | B | | X |  | | CL; CM-Pf; TRN; MD; LP; PUL; Lateral geniculate body | | |  |
| Yu et al. (130) | | 2021 | Consciousness | | Human | | 10 | Mixed | | X |  | | MD; VA | | |  |
| Kondratyeva et al. (199) | | 2020 | Consciousness | | Human | | 34 | B | | X |  | | PULm; PULl | | |  |
| Kumral et al. (89) | | 2015 | Consciousness | | Human | | 252 | B | |  | X | | CL; CM-Pf; TRN; MD; LP; PUL; Lateral geniculate body | | |  |
| Laouchedi et al. (95) | | 2015 | Consciousness | | Human | | 24 | B | |  | X | | CM-Pf; TRN; VA; VL; VP; PUL; MD | | |  |
| Feng et al. (111) | | 2017 | Consciousness | | Animal | | 38 | - | |  | X | | CL; VPM | | |  |
| *Anteroventral (Av)* | | | | | | | | | | | | | | | | |
| Weiner et al. (33) | | 2023 | Consciousness | | Human | | 11 | B | | X |  | | MD; PULl; PULa; PULi; PULm; VPL; Sg-Li; LP; LD; CM-Pf; VLp; VLa; Re; CeM; VAmc; VA; CL; Medial geniculate body; Lateral geniculate body | | |  |
| Claar et al. (106) | | 2023 | Consciousness | | Animal | | - | - | | X |  | | CL; TRN; MD; Po; VA; VPL; VPM | | |  |
| Setzer et al. (100) | | 2022 | Arousal | | Human | | 27 | - | |  | X | | CM-Pf; VPL; MD; PUL; VA; VPL; VLa; VLp; Lateral geniculate body | | |  |
| *Anterodorsal (Ad)* | | | | | | | | | | | | | | | | |
| Gent et al. (124) | 2018 | | | Wakefulness | | Animal | - | | B | X | |  | | CeM; Re; Pv; VP; MD |  | |

# Supplementary Table S9

***Supplementary Table S9***

**Supplementary Table S9.** The table lists the studies providing evidence of a relationship between consciousness/arousal/wakefulness and the posterior nuclear group.

Ad: anterodorsal nucleus; Av: anteroventral nucleus; CeM: central medial nucleus; CL: central lateral nucleus; CM-Pf: centromedian-parafascicular-complex; LD: laterodorsal; LP: lateral posterior; MD: mediodorsal nucleus; MDmc: mediodorsal magnocellular part; MDpc: mediodorsal parvocellullar parts; MDpl: mediodorsal paralaminar part; Pc: paracentral nucleus; Po: posterior nucleus; Pt: paratenial; PUL: pulvinar nucleus; PULa: pulvinar anterior part; PULi: pulvinar inferior part; PULl: pulvinar lateral part; PULm: pulvinar medial part; Pv: paraventricular; Re: reuniens; Sg-Li: suprageniculates-limitans nucleus; TRN: reticular nucleus; VA: ventral anterior nucleus; VL: ventral lateral nucleus; VLa: ventral lateral anterior part; VLp: ventral lateral posterior part; VP: ventral posterior complex; VPL: ventral posterolateral nucleus; VPM: ventral posteromedial nucleus.

| **Authors** | | **Year** | **Function** | | **Population** | | **Sample size** | | **Lateralization** | | | **Evidence** | | | **Other nuclei** | **Notes** |
| --- | --- | --- | --- | --- | --- | --- | --- | --- | --- | --- | --- | --- | --- | --- | --- | --- |
|  |  |  |  |  |  |  |  |  |  |  |  | **Positive** | **Negative** | |  |  |
| Sukhotinsky et al. (97) | | 2007 | Consciousness | | Animal | | - | | B | | | X |  | | CM-Pf; CL; Pc; VL; A; TRN; CeM; Midline nuclear group |  |
| Flores et al. (112) | | 2017 | Consciousness | | Animal | | - | | - | | | X |  | | CL; LD; MD |  |
| Tenney et al. (164) | | 2003 | Consciousness | | Animal | | 8 | | B | | | X |  | | VPM; VPL; TRN; LD |  |
| Alkire et al. (120) | | 2009 | Consciousness | | Animal | | 106 | | - | | |  | X | | CeM; VL |  |
| *Suprageniculate – Limitans (Sg-Li)* | | | | | | | | | | | | | | | | |
| Weiner et al. (33) | 2023 | | | Consciousness | | Human | | 11 | | B | X | | |  | MD; PULl; PULa; PULi; PULm; VPL; LP; LD; Av; CM-Pf; VLp; VLa; Re; CeM; VAmc; VA; CL; Medial geniculate body; Lateral geniculate body |  |
| *Posterior (Po)* | | | | | | | | | | | | | | | | |
| Claar et al. (106) | | 2023 | Consciousness | | Animal | | - | | - | | | X |  | | CL; TRN; Av; MD; VA; VPL; VPM |  |

# Supplementary Table S10

***Supplementary Table S10***

**Supplementary Table S10.** The table lists the studies providing evidence of a relationship between consciousness/arousal/wakefulness and the medial geniculate body.

Ad: anterodorsal nucleus; Av: anteroventral nucleus; CeM: central medial nucleus; CL: central lateral nucleus; CM-Pf: centromedian-parafascicular-complex; LD: laterodorsal; LP: lateral posterior; MD: mediodorsal nucleus; MDmc: mediodorsal magnocellular part; MDpc: mediodorsal parvocellullar parts; MDpl: mediodorsal paralaminar part; Pc: paracentral nucleus; Po: posterior nucleus; Pt: paratenial; PUL: pulvinar nucleus; PULa: pulvinar anterior part; PULi: pulvinar inferior part; PULl: pulvinar lateral part; PULm: pulvinar medial part; Pv: paraventricular; Re: reuniens; Sg-Li: suprageniculates-limitans nucleus; TRN: reticular nucleus; VA: ventral anterior nucleus; VL: ventral lateral nucleus; VLa: ventral lateral anterior part; VLp: ventral lateral posterior part; VP: ventral posterior complex; VPL: ventral posterolateral nucleus; VPM: ventral posteromedial nucleus.

| **Authors** | **Year** | **Function** | **Population** | **Sample size** | **Lateralization** | **Evidence** | | **Other nuclei** | **Notes** |
| --- | --- | --- | --- | --- | --- | --- | --- | --- | --- |
|  |  |  |  |  |  | **Positive** | **Negative** |  |  |
| Weiner et al. (33) | 2023 | Consciousness | Human | 11 | B | X |  | MD; PULl; PULa; PULi; PULm; VPL; Sg-Li; LP; LD; Av; CM-Pf; VLp; VLa; Re; CeM; VAmc; VA; CL; Lateral geniculate body |  |
| Andrada et al. (154) | 2012 | Consciousness | Animal | 5 | - | X |  | VPL; VPM |  |
| Lutkenhoff et al. (34) | 2013 | Consciousness | Human | 25 | B |  | X | LD; MD; VA; VPL; VL; VPM; LP; PUL; Lateral geniculate body; Anterior nuclear group |  |

# Supplementary Table S11

***Supplementary Table S11***

**Supplementary Table S11.** The table lists the studies providing evidence of a relationship between consciousness/arousal/wakefulness and the lateral geniculate body.

Ad: anterodorsal nucleus; Av: anteroventral nucleus; CeM: central medial nucleus; CL: central lateral nucleus; CM-Pf: centromedian-parafascicular-complex; LD: laterodorsal; LP: lateral posterior; MD: mediodorsal nucleus; MDmc: mediodorsal magnocellular part; MDpc: mediodorsal parvocellullar parts; MDpl: mediodorsal paralaminar part; Pc: paracentral nucleus; Po: posterior nucleus; Pt: paratenial; PUL: pulvinar nucleus; PULa: pulvinar anterior part; PULi: pulvinar inferior part; PULl: pulvinar lateral part; PULm: pulvinar medial part; Pv: paraventricular; Re: reuniens; Sg-Li: suprageniculates-limitans nucleus; TRN: reticular nucleus; VA: ventral anterior nucleus; VL: ventral lateral nucleus; VLa: ventral lateral anterior part; VLp: ventral lateral posterior part; VP: ventral posterior complex; VPL: ventral posterolateral nucleus; VPM: ventral posteromedial nucleus.

| **Authors** | **Year** | **Function** | **Population** | **Sample size** | **Lateralization** | **Evidence** | | **Other nuclei** | **Notes** |
| --- | --- | --- | --- | --- | --- | --- | --- | --- | --- |
|  |  |  |  |  |  | **Positive** | **Negative** |  |  |
| Weiner et al. (33) | 2023 | Consciousness | Human | 11 | B | X |  | MD; PULl; PULa; PULi; PULm; VPL; Sg-Li; LP; LD; Av; CM-Pf; VLp; VLa; Re; CeM; VAmc; VA; CL; Medial geniculate body |  |
| Setzer et al. (100) | 2022 | Arousal | Human | 27 | - | X |  | CM-Pf; VPL; Av; MD; PUL; VA; VPL; VLa; VLp |  |
| Edlow et al. (52) | 2013 | Consciousness | Human | 1 | B |  | X | CL; CM-Pf; TRN; MD; LP; PUL; Anterior nuclear group |  |
| Lutkenhoff et al. (34) | 2013 | Consciousness | Human | 25 | B |  | X | LD; MD; VA; VPL; VL; VPM; LP; PUL; Lateral geniculate body; Medial geniculate body; Anterior nuclear group |  |
